# A Complete Survey of RhoGDI Targets Reveals Novel Interactions with Atypical Small GTPases

**DOI:** 10.1021/acs.biochem.1c00120

**Published:** 2021-04-29

**Authors:** Ana Masara binti Ahmad Mokhtar, Samrein B. M. Ahmed, Nicola J. Darling, Matthew Harris, Helen R. Mott, Darerca Owen

**Affiliations:** Department of Biochemistry, University of Cambridge, 80 Tennis Court Road, Cambridge CB2 1GA, United Kingdom

## Abstract

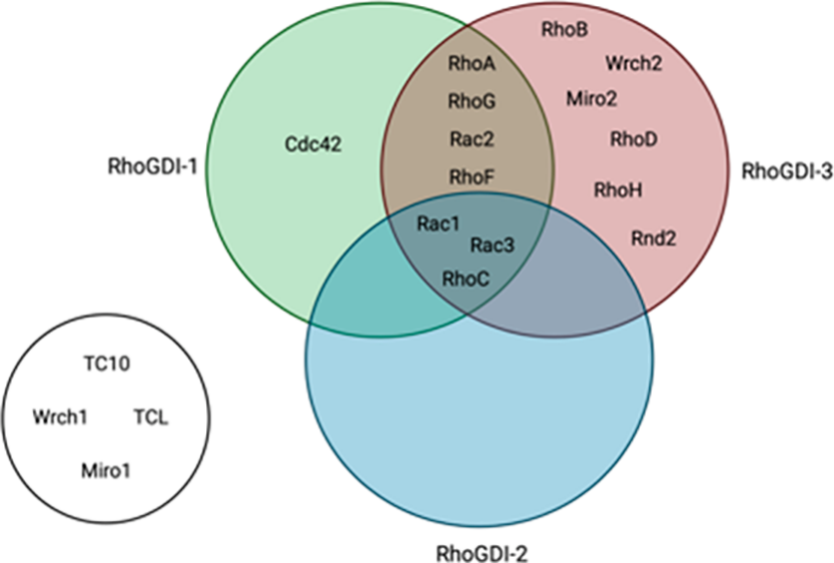

There are three RhoGDIs in mammalian cells, which were initially defined as negative regulators of Rho family small GTPases. However, it is now accepted that RhoGDIs not only maintain small GTPases in their inactive GDP-bound form but also act as chaperones for small GTPases, targeting them to specific intracellular membranes and protecting them from degradation. Studies to date with RhoGDIs have usually focused on the interactions between the “typical” or “classical” small GTPases, such as the Rho, Rac, and Cdc42 subfamily members, and either the widely expressed RhoGDI-1 or the hematopoietic-specific RhoGDI-2. Less is known about the third member of the family, RhoGDI-3 and its interacting partners. RhoGDI-3 has a unique N-terminal extension and is found to localize in both the cytoplasm and the Golgi. RhoGDI-3 has been shown to target RhoB and RhoG to endomembranes. In order to facilitate a more thorough understanding of RhoGDI function, we undertook a systematic study to determine all possible Rho family small GTPases that interact with the RhoGDIs. RhoGDI-1 and RhoGDI-2 were found to have relatively restricted activity, mainly binding members of the Rho and Rac subfamilies. RhoGDI-3 displayed wider specificity, interacting with the members of Rho, Rac, and Cdc42 subfamilies but also forming complexes with “atypical” small Rho GTPases such as Wrch2/RhoV, Rnd2, Miro2, and RhoH. Levels of RhoA, RhoB, RhoC, Rac1, RhoH, and Wrch2/RhoV bound to GTP were found to decrease following coexpression with RhoGDI-3, confirming its role as a negative regulator of these small Rho GTPases.

Small GTPases, comprising the Ras superfamily, are monomeric guanine nucleotide binding proteins. The Ras superfamily can be divided into five major families: Ras, Rho, Arf, Ran, and Rab. Although each small G protein has a distinct molecular sequence and cellular function, they all share a basic conserved guanine nucleotide binding domain (the G domain) and mostly utilize a shared conformational switching ability in order to function.^1^

The Rho family small GTPases are best studied for their role in promoting actin cytoskeletal reorganization; however, they also have roles in cell division, cell adhesion and motility, vesicular trafficking, phagocytosis, and transcriptional regulation.^2^ Their defining feature is the Rho insert region, located between the fifth β-strand and the fourth α-helix in the G domain.^3^ In humans, there are 20 Rho family members that can be further categorized into eight subfamilies based on their amino acid sequence identity, structural motifs, and biological functions (Figure 1A,B and Table S1). The Miro (mitochondrial Rho) proteins are related but are now considered to form separate branches of the Ras superfamily.^4^

**Figure 1:**
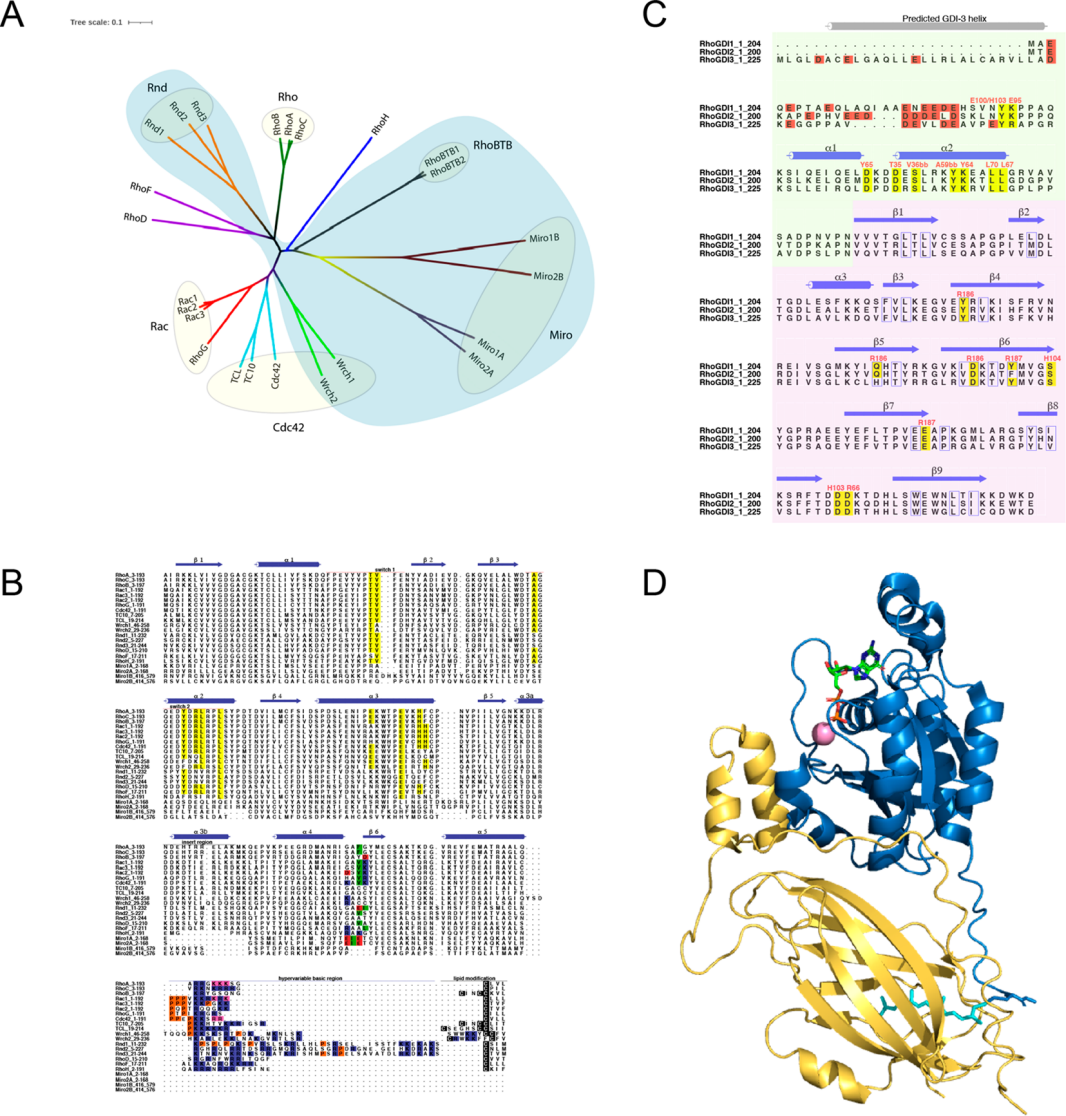
(A) Phylogenetic tree of the Rho-family GTPases. An alignment of all Rho family small G proteins and the first G domain of the Miro proteins was extracted from Rojas et al.^67^ A secondary structure mask was added for RhoA, together with the second G domain of the Miro protein, in ClustalX in profile alignment mode.^68^ This alignment was uploaded into iTOL v7^69^ to create the tree. Atypical Rho family GTPases appear on a blue background. (B) Sequence alignment of the Rho family. Sequence alignment of the Rho family (except the RhoBTB proteins) and the two G domains in Miro1 and Miro2 (labeled A and B). The C-terminus of the Rho family proteins is too variable to reliably align, so the first basic residues of all the proteins were aligned and the isoprenylated cysteines were aligned in a separate block with all other C-terminal Cys residues. The secondary structural elements are depicted above the alignment as blue cylinders (α-helices) or arrows (β-strands). Residues that interact with RhoGDI-1 in the Cdc42 complex (PDB code 1doa) are colored yellow in Cdc42 and in all family members that are identical. Pro residues in the C-terminus are colored orange, and the basic residues are colored blue, except the dibasic motifs in RhoA, Rac1, and Cdc42 that have been shown to bind to RhoGDIs. The Cys residues at the extreme C-terminus that are assumed to be lipid modified are shown in white on a black background. Residues in the divergent loop between helix α4 and strand β5 that may be partially responsible for RhoGDI-3 discrimination are colored red (acidic); blue (basic); and green (large hydrophobic). (C) Sequence alignment of the three human RhoGDI proteins. The secondary structural elements are depicted above the alignment as blue cylinders (α-helices) or arrows (β-strands). The position of the α-helix predicted at the N-terminus of RhoGDI-3 is shown in gray. The acidic residues in the N-terminal extension of all the GDIs are colored red. Residues that interact with the geranylgeranyl group in the Cdc42–RhoGDI-1 structure (PDB code1doa) are boxed in blue. Residues that interact with the Cdc42 protein moiety are colored yellow in RhoGDI-1 and the other two members of the family if they are conserved. The Cdc42 residues with which they interact are above each one, where “bb” indicates that it is the backbone of the Cdc42 residue that is involved in the interaction. The N-terminal region of the RhoGDIs is highlighted in a pale lime box and the C-terminal Ig domain in a pale mauve box. (D) Structure of RhoGDI-1 in complex with Cdc42 (PDB code 1doa). RhoGDI-1 is shown in yellow and Cdc42 is shown in blue. The geranylgeranyl at the C-terminus of Cdc42 is in cyan. The nucleotide is shown in a stick representation with carbons in green, oxygens in red, nitrogens in blue, and phosphoruses in orange. The Mg^2+^ ion is shown as a pale pink sphere.

The most extensively studied Rho family members are RhoA, Rac1, and Cdc42. These proteins and their subfamilies are also known as classical or typical Rho GTPases as they cycle between GDP-bound inactive and GTP-bound active states in the cell.^5^ The other group of Rho family GTPases is known as the atypical Rho GTPases and includes, for example, the RhoBTB, Wrch, and Rnd subfamilies (Figure 1A). These often contain extra domains or short N-terminal and C-terminal extensions making them larger than the classical Rho GTPases. For example, the RhoBTB subfamily (comprising RhoBTB1 and RhoBTB2) contains two extra BTB (broad complex, Tramtrack, and Bric-a-brac) domains. RhoBTB subfamily members lack a CAAX motif at their C-termini, which usually directs post-translational isoprenylation in small G proteins.^5^ Most Rho GTPases undergo C-terminal lipid modification, usually geranylgeranylation or farnesylation and, less frequently, palmitoylation, all of which allow them to associate with membranes where they exert their biological functions.^6^ The Miro family proteins have an additional GTPase domain and two EF-hand motifs and are not lipid modified at their C-termini.

The classic Rho family GTPases cycle between an inactive, GDP-bound state and an active, GTP-bound state and therefore act as conventional biological binary switches in line with most other members of the Ras superfamily. Their activation status is highly dependent upon three classes of regulatory proteins known as guanine nucleotide exchange factors (GEFs), GTPase-activating proteins (GAPs), and guanine nucleotide dissociation inhibitors (GDIs). GEFs facilitate GDP dissociation and promote binding of the more abundant GTP from the cytoplasm, thus allowing activated small GTPases to bind to their specific effector proteins and trigger the corresponding signaling pathways.^7^ In contrast, GAPs are responsible for terminating small GTPases signaling by stimulating their intrinsic GTPase activity and enhancing the hydrolysis of GTP to GDP.^7^ GDIs are bifunctional, negative regulators for small GTPases: they bind to the switch regions of the G proteins, preventing nucleotide exchange, and also physically sequester small GTPases from membranes holding them in their inactive form in the cytosol.^8,9^ Importantly RhoGDIs also act to couple the nucleotide cycle of the Rho family GTPases to a membrane cycle, with the small G proteins being localized to their appropriate membrane for signaling or held in the cytoplasm.^10^

In contrast, the atypical Rho family GTPases are thought to exist primarily in the GTP-bound form as they rarely follow the common GTP–GDP cycle described above. In accordance with this, these proteins appear to be regulated by other means. For example, Rnd protein function is negatively regulated by 14–3–3 proteins^11^ and ubiquitin/proteasome degradation,^12^ while phosphorylation of Rnd proteins can affect their localization and protect them from degradation.^13^ RhoH function has been found to be regulated by expression, and alteration of RhoH transcription level has also been shown to affect the activities of other Rho GTPases such as Rac1, RhoA, and Cdc42 by suppressing the activation of NF-κB.^14^ Since these atypical Rho GTPases are constitutively GTP-bound, the argument follows that they are not targets for RhoGDIs. However, RhoH does form a complex with all three RhoGDIs *in vivo*,^14^ suggesting that RhoGDI complexes are not restricted to the GDP-form of Rho family proteins and that RhoGDIs also have a role in regulating the activity of the atypical Rho GTPases.

There are a total of 145 RhoGEFs and RhoGAPs in mammalian cells but only three RhoGDIs (Figure 1C) and their role is still not fully understood.^15^ There is growing evidence that RhoGDIs are not only negative regulators of Rho GTPases but also act as chaperones to target the Rho GTPases to specific subcellular compartments such as the Golgi.^10,16^ The chaperone function of the RhoGDIs also stabilizes the Rho proteins and prevents them from being targeted for proteosomal degradation.^17^ RhoGDI-1 depletion has been found to increase the accumulation of newly synthesized Rho family proteins in the endoplasmic reticulum, where post-translational modification occurs, again indicating a role for RhoGDI-1 as a chaperone.^17^ Besides functioning to stabilize the inactive GDP-bound form of the small GTPases, RhoGDIs have also been shown to be necessary for maintaining the GTP-bound state of certain small GTPases. For instance, the RhoGDI–Cdc42·GTP interaction is needed for Cdc42-induced cell proliferation and transformation.^18^ The RhoGDI-1–Rac1 interaction is also crucial for stimulating the NADPH oxidase system in neutrophils.^19^ In contrast, the RhoGDI-1–Rac2 complex is found to abrogate the activation of NADPH oxidase,^20^ suggesting target-specific functions, at least for RhoGDI-1.

The interaction between the RhoGDIs and the Rho GTPases involves both major domains of the RhoGDIs; the N-terminal regulatory arm region and the C-terminal immunoglobulin-like domain (Figure 1C,D). Both regions contribute significantly to the binding and inhibitory actions of the RhoGDI proteins. The C-terminal Ig domain of the RhoGDIs forms a hydrophobic cleft that binds to the geranylgeranyl group at the C-termini of the Rho family proteins thus preventing membrane association (Figure 1D). The N-terminal region of the RhoGDIs is intrinsically disordered in the free form^21,22^ but undergoes a disorder–order transition upon binding to its Rho family targets, with a newly structured helical hairpin binding to the switch region of the target small GTPases, inhibiting nucleotide exchange^8^ (Figure 1D). All three RhoGDIs share the same domain architecture; however, RhoGDI-3 has an N-terminal extension containing a putative amphipathic helix that plays an important role in Golgi targeting and stabilizing the cytoplasmic RhoG–RhoGDI-3 complex^16^ (Figure 1C). RhoGDI-1 and RhoGDI-2 share 68% sequence identity, whereas RhoGDI-3 shows 55–57% identity with RhoGDI-1 and RhoGDI-2 (Figure 1C).

Previous assessments summarizing the information correlating RhoGDIs with their target proteins reveal that a broad overview is lacking.^23^ In this work we have made a systematic, comprehensive study of the three RhoGDI proteins and their binding profiles to all Rho family proteins and to the related RhoBTB and Miro families. These data should help to elucidate the functional differences between the three RhoGDIs. We confirm previously identified interactions and add interesting new Rho family G protein interactions with the RhoGDI family, especially with the atypical Rho GTPases. We also present an analysis of the binding profiles of the RhoGDI proteins in terms of the structural data available, in order to define, assess, and understand consensus contacts in the RhoGDI–G protein complexes.

## Materials and Methods

### Cell Lines and DNA Constructs

Human embryonic kidney HEK293T cells were maintained in Dulbecco’s modified Eagle’s medium (DMEM) containing 4.5 g/L glucose, supplemented with 10% foetal bovine serum (FBS) (Merck), 2 mM l-glutamine (Merck), and 1% antibiotic-antimycotic (Merck) in a 37 °C humidified incubator supplemented with 5% CO_2_. Full-length human RhoGDI-1, RhoGDI-2, and RhoGDI-3 cDNAs and cDNAs of all 23 small GTPases were amplified by PCR, cloned into pENTR/D-TOPO (Thermo Fisher Scientific), and then transferred into the mammalian expression vectors pDEST12.2-FLAG, pcDNA3.1-nV5, and pDEST26 using Gateway technology, following the manufacturer’s instructions (ThermoFisher Scientific). All constructs produced N-terminally tagged fusion proteins. Transient transfection of each RhoGDI alone or in the presence of Rho family small GTPases was performed using Lipofectamine reagent (Life Technologies), according to the manufacturer’s instructions, or polyethylenimine (PEI; DNA was mixed with 30 μL of 1 mg/mL PEI and 1 mL of DMEM and left at room temperature (RT) for 10 min, before being added dropwise to cells) for 40 h with a minimum of 0.5 μg of GFP DNA as a transfection control. After 40 h, cells were lysed and subjected to coimmunoprecipitation and Western blotting.

### Coimmunoprecipitation

Transfected cells were lysed using cold mammalian lysis buffer [50 mM Tris-HCl pH 7.4, 150 mM NaCl, 1 mM EDTA, 1 mM Na_3_VO_4_, 1 mM β-glycerophosphate disodium salt hydrate, 1× mammalian protease inhibitor complex (Sigma), and 1% Triton X-100] and pelleted at 13 000*g* for 20 min. RhoGDIs were precipitated with protein G beads (Merck) conjugated to 1 mg/mL anti-FLAG (ThermoFisher Scientific) or nickel-coated beads (Merck) according to the manufacturer’s instruction. Immunoprecipitated proteins were then eluted in NuPAGE LDS sample buffer (ThermoFisher Scientific) containing 2.3 M β-mercaptoethanol. Bound Rho-family small GTPases were determined by Western blot analysis.

### Western Blotting

All expression trials and coimmunoprecipitation samples were separated using NuPAGE 4–12% SDS-PAGE gels. The proteins were then transferred to an Immobilon-P PVDF membrane using an XCell II blot module. After transfer, membranes were stained with PonceauS for 1 min to check the efficiency of protein transfer before blocking with 10% skimmed milk at 4 °C for a minimum of 1 h. Next, the membrane was incubated with anti-His-HRP (sc-8036 HRP, Santa Cruz) for His-tagged RhoGDI-1 and RhoGDI-2, anti-FLAG-HRP (A8592, Sigma-Aldrich) for FLAG-tagged RhoGDI-3, anti-V5-HRP (R961-25, ThermoFisher Scientific) for all 23 small GTPases, anti-Rac1 (05-389, Merck), and anti-GAPDH-HRP (ab9482, Abcam). Proteins were visualized by treating with enhanced chemiluminescence solution for 2 min and exposing the membrane to medical X-ray film (Konica).

### Protein Expression and Purification

GST-human PAK1-PBD^24^ was expressed in *Escherichia coli* BL21 at 37 °C for 5 h and purified using glutathione-agarose beads (Sigma). The amount of protein was quantified using A_280_. pGEX-2T-Rhotekin (1–89) was expressed in *E. coli* BL21 (DE3) pLysS at 20 °C, overnight. GST-fused Rhotekin was then purified using glutathione-sepharose 4B beads (GE Healthcare) and stored at −80 °C as a 5% suspension in 50 mM Tris-HCl, pH 7.5, 150 mM NaCl, 5 mM MgCl_2_, and 10% glycerol.

### Effector Pull Down

GST-PAK1 PBD was used as an effector for Rac1, RhoH, and Wrch2/RhoV. HEK293T cells were transfected with the relevant expression construct and lysed after 40 h in buffer (20 mM Tris-HCl pH 7.5, 150 mM NaCl, 5 mM MgCl_2_, 5 mM β-glycerophosphate, 1 mM DTT) containing 10 μg of GST-PAK1 PBD. The lysate was then incubated with glutathione-sepharose 4B beads for 45 min at 4 °C. The beads were washed with lysis buffer and resuspend in LDS sample buffer. GTP-bound small GTPases were identified by Western blotting using antibodies specific for Rac1 (05-389, Merck) or anti-V5 (R961-25, Invitrogen) for RhoH and Wrch2/RhoV.

GST-Rhotekin 1–89 was used as an effector for RhoA, RhoB, and RhoC. Transfected HEK293T cells were lysed as above, prior to incubation with 100 μL (10 μg) of GST-Rhotekin-bead suspension for 45 min at 4 °C. Following incubation, the beads were washed and eluted with LDS sample buffer. GTP-bound RhoA was identified by Western blotting with anti-RhoA (26C4, sc-418, Santa Cruz Biotechnology Inc.); GTP-bound RhoB with anti-RhoB (14326-1-AP, Proteintech); and GTP-bound RhoC with anti RhoC (D40E4, 3430S, Cell Signaling Technology).

### Modeling

The RhoGDI proteins were aligned using TM-COFFEE. A model of Cdc42 with RhoGDI-3 starting at residue 31 was generated using MODELLER9.2.^25^ The secondary structure prediction using Jpred indicated a helix from residues 6–26 in RhoGDI-3, and this was built using Avogadro.^26^ Residues 27–30 were built onto the N-terminus of RhoGDI-3 in Pymol and the extra helix was added manually. This was then refined using the YASARA energy minimization server and the RhoGDI-3 component extracted from the model. The models of the complex were built using MODELLER9.2 and the structures of Cdc42–RhoGDI-1 (PDB code 1doa), Rac1–RhoGDI-1 (PDB code 1hh4) and the structure of the Rho family protein in its free form if it existed: for RhoB, PDB code 2fv8, and for RhoD, PDB code 2j1l. For Wrch2/RhoV, the structure of Wrch1/RhoU (PDB code 2q3h) was used, and the Cdc42–RhoGDI-1 model included a palmitoyl instead of geranylgeranyl. This was generated by overlaying the Cys from high-resolution crystal structures of palmitoylated Cys-containing proteins with the geranylgeranylated Cys from Cdc42 in Pymol. The only palmitoyl group that was able to fit into the isoprenyl pocket without obvious clashes was that of the TEAD2 transcription factor (PDB code 5emv). All the models were built with loop refinement using the DOPE-based loop modeling protocol. The best model for each structure (with the lowest MODELER objective function) was analyzed using CCP4 Contact to find the interactions between the two components of the complex. Interactions between residues that were less than 4 Å and where two or more contacts were seen were used to generate an ambiguous interaction restraint list for HADDOCK 2.4.^27^ Each complex was then put into HADDOCK 2.4, where the initial orientation of the complex was maintained, but the entire N-terminus of RhoGDI-3 (residues 6–37) and the C-terminus of the Rho protein beyond the helix α5 (except the modified Cys) were defined as fully flexible. The models were refined in implicit water, which was followed by short molecular dynamics in explicit water. Of the top 4 structures generated by HADDOCK, the one where the N-terminal helix was longest was selected.

## Results

### RhoGDI-1 Binding to Classical Rho Family Members

RhoGDI-1 is ubiquitously expressed and has been shown previously to form complexes with RhoA, RhoC, Rac1, Rac2, Cdc42, and RhoG.^28,29^ All of the Rho family small GTPases identified as RhoGDI-1 targets to date are known regulators of actin cytoskeletal reorganization and also play crucial roles in controlling cell proliferation and migration.^30^

**Figure 2:**
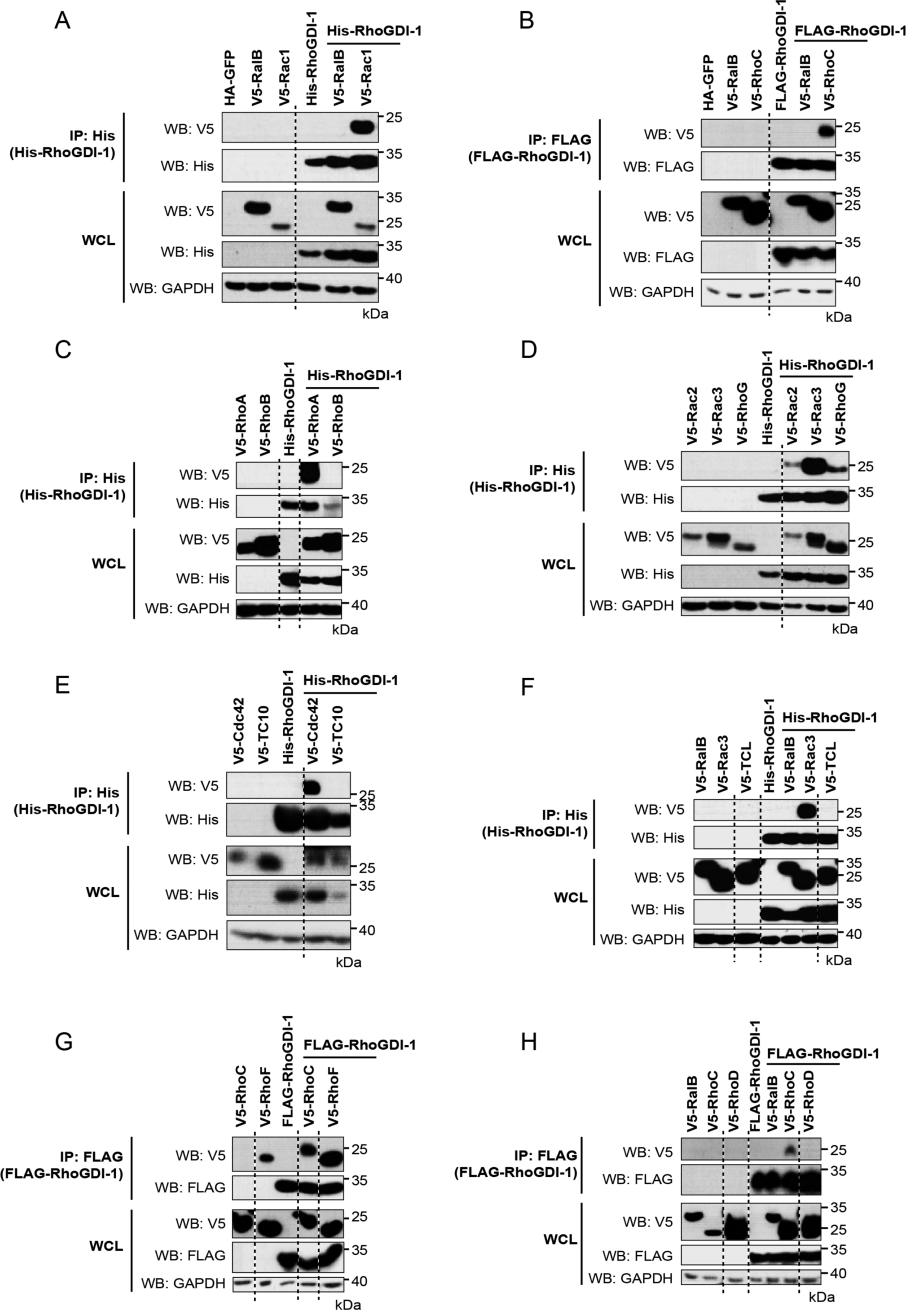
Interaction between RhoGDI-1 and the classical Rho GTPases. V5-tagged constructs of the 12 classical Rho GTPases were expressed alone and with His-tagged RhoGDI-1. Expression of the recombinant proteins in whole cell lysate (WCL) is shown in the bottom panels. RhoGDI-1 was precipitated from cell lysates using nickle-coated magnetic beads, and the coimmunoprecipitation of the Rho GTPases was assessed using an anti-V5 antibody (top panels). Results are representative of at least three independent experiments. (A) Rac1; (B) RhoC; (C) RhoA and RhoB; (D) Rac2, Rac3, and RhoG; (E) Cdc42 and TC10/RhoQ; (F) TCL/RhoJ; (G) RhoF; and (H) RhoD.

His-tagged or FLAG-tagged RhoGDI-1 were coexpressed with V5-tagged versions of all 12 classical members of the Rho family and RalB was included as a negative control. Lysates were then incubated with nickel-coated or anti FLAG-coated beads and the precipitated proteins analyzed by Western blotting. RhoGDI-1 was seen to interact with Rac1 (Figure 2A); RhoA (Figure 2C); Rac2, Rac3, and RhoG (Figure 2D); and Cdc42 (Figure 2E). These data confirm the interaction profile already established for RhoGDI-1 and introduce Rac3 as a RhoGDI-1 partner for the first time. RhoGDI-1 failed to interact with RhoB (Figure 2C). Despite binding to Cdc42, RhoGDI-1 did not interact with the other classical members of the Cdc42 subfamily, TC10/RhoQ (Figure 2E) and TCL/RhoJ (Figure 2F). RhoGDI-1 was unable to bind to RhoD (Figure 2H) but likely interacts with RhoF (Figure 2G), which is in the same subfamily.

### RhoGDI-2 Displays Increased Selectivity in Its Classical Rho Family Target Profile

The second member of the family, RhoGDI-2 (also known as D4/Ly-GDI), is selectively expressed at high levels in hematopoietic cells such as B and T lymphocytes.^31^ Although the full target profile for RhoGDI-2 is yet to be defined, it has been shown to bind to Rac2, RhoA, RhoC, and, to lesser extents, Rac1, Cdc42, and RhoG.^32,33^

**Figure 3:**
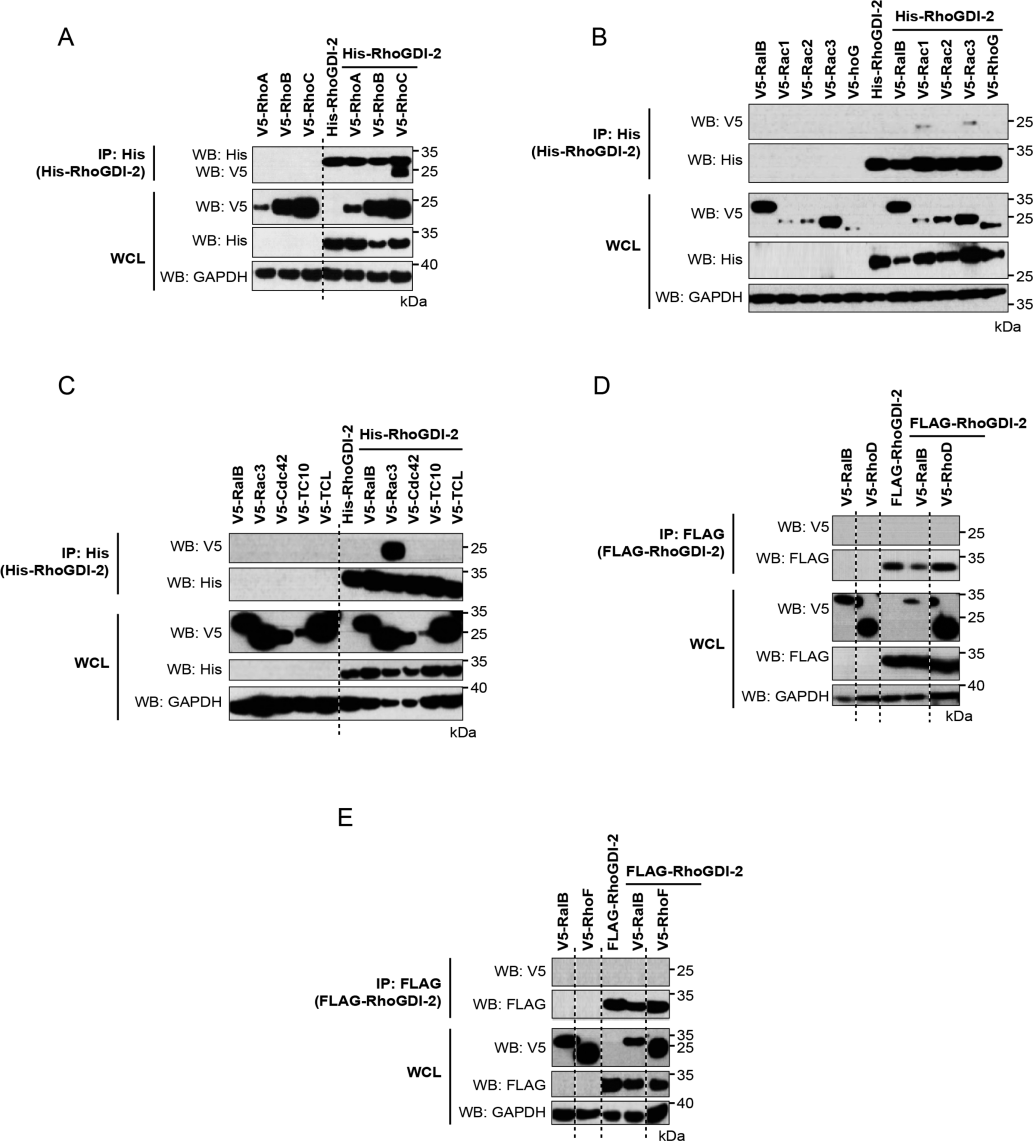
Interaction between RhoGDI-2 and the classical Rho GTPases. V5-tagged constructs of the 12 classical Rho GTPases were expressed alone and with His-tagged RhoGDI-2. Expression of the recombinant proteins in whole cell lysate (WCL) is shown in the bottom panels. RhoGDI-2 was precipitated from cell lysates using nickle-coated magnetic beads, and the coimmunoprecipitation of the Rho GTPases was assessed using an anti-V5 antibody (top panels). Results are representative of at least three independent experiments. (A) RhoA, RhoB, and RhoC; (B) Rac1, Rac2, Rac3, and RhoG; (C) Cdc42, TC10/RhoQ, and TCL/RhoJ; (D) RhoD; and (E) RhoF.

His-tagged RhoGDI-2 were tested for binding V5-tagged Rho family members as described above. RhoGDI-2 was only found to interact with RhoC, Rac1, and Rac3 (Figure 3A,B). No binding was seen with RhoB (Figure 3A), TC10/RhoQ, TCL/RhoJ (Figure 3C), RhoD (Figure 3D), or RhoF (Figure 3E). Binding was also not observed for RhoA (Figure 3A), Cdc42 (Figure 3C), Rac2, or RhoG (Figure 3B) in this system, despite being observed in previous studies.^32,33^

### RhoGDI-1 and RhoGDI-2 Binding to Atypical Small RhoGTPases and Miro Proteins

**Figure 4:**
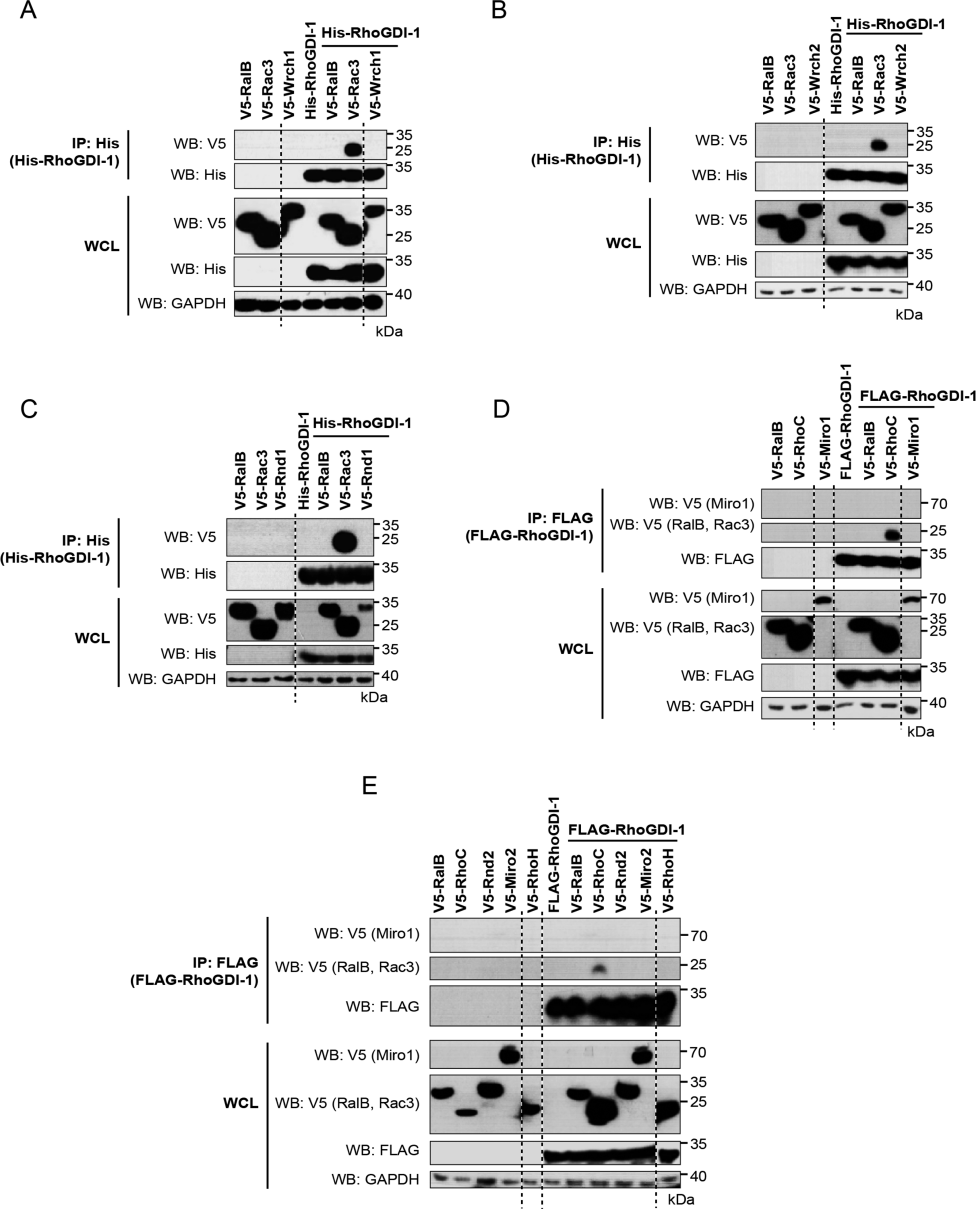
Interaction between RhoGDI-1 and the atypical Rho GTPases. V5-tagged constructs of the 11 atypical Rho GTPases were expressed alone and with His-tagged or FLAG-tagged RhoGDI-1. Expression of the recombinant proteins in whole cell lysate (WCL) is shown in the bottom panels. RhoGDI-1 was precipitated from cell lysates using nickle-coated magnetic beads or immunoprecipitated using anti-FLAG antibody cross-linked to magnetic beads. The coimmunoprecipitation of the Rho GTPases was assessed using an anti-V5 antibody (top panels). Results are representative of at least three independent experiments. (A) Wrch1/RhoU, (B) Wrch2/RhoV, (C) Rnd1, (D) Miro1, and (E) Rnd2, Miro2, and RhoH.

**Figure 5:**
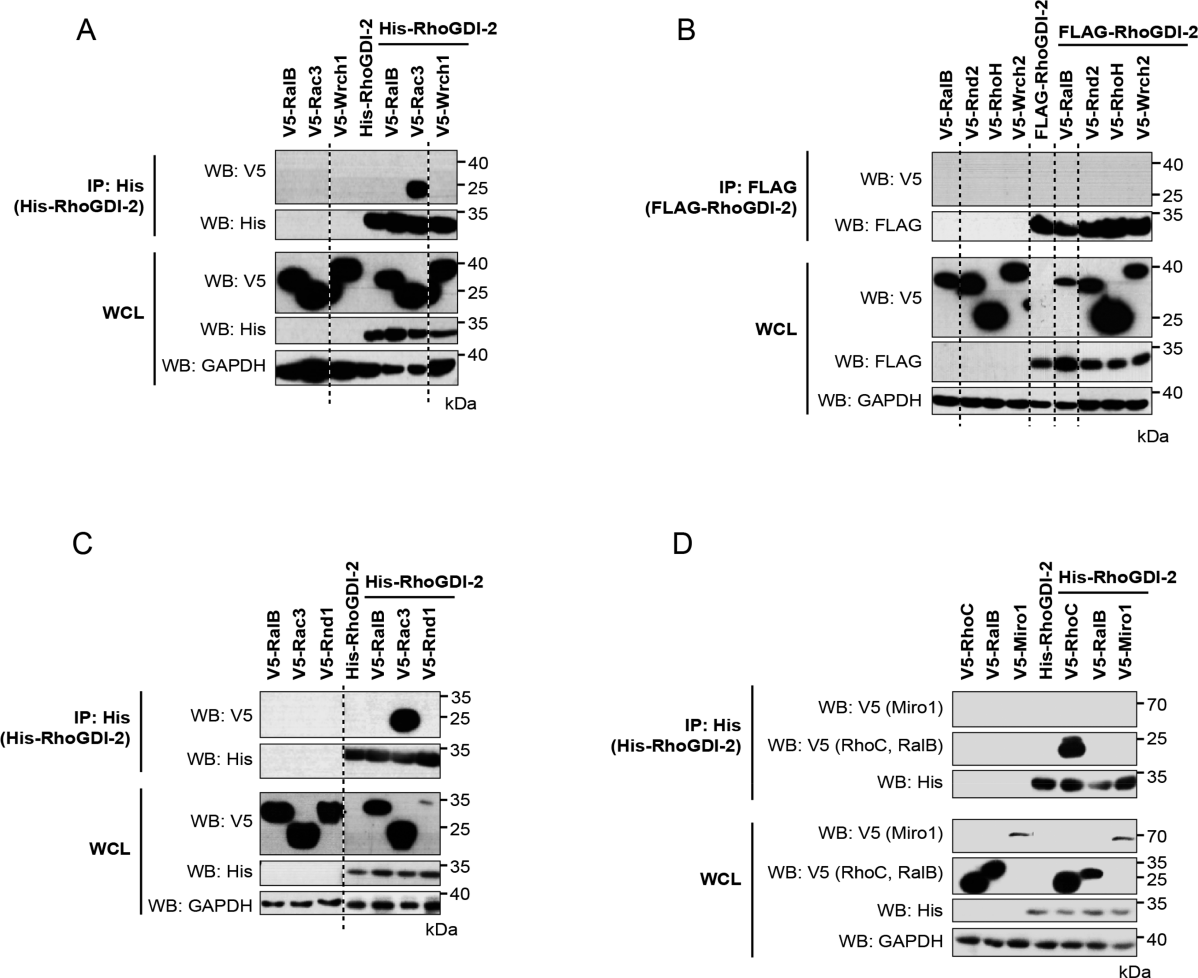
Interaction between RhoGDI-2 and the atypical Rho GTPases. V5-tagged constructs of the 11 atypical Rho GTPases were expressed alone and with His-tagged or FLAG-tagged RhoGDI-2. Expression of the recombinant proteins in whole cell lysate (WCL) is shown in the bottom panels. RhoGDI-2 was precipitated from cell lysates using nickle-coated magnetic beads or immunoprecipitated using anti-FLAG antibody cross-linked to magnetic beads. The coimmunoprecipitation of the Rho GTPases was assessed using an anti-V5 antibody (top panels). Results are representative of at least three independent experiments. (A) Wrch1/RhoU; (B) Rnd2/RhoN, RhoH, and Wrch2/RhoV; (C) Rnd1; and (D) Miro1.

Very few studies have examined the binding of the RhoGDIs to the atypical Rho GTPases. Previously, however, Li et al. have reported that all three RhoGDIs can interact with RhoH.^14^ His-tagged or FLAG-tagged RhoGDI-1 and RhoGDI-2 were coexpressed with V5-tagged versions of all 8 atypical Rho family GTPases. Coexpression trials were carried out to determine the appropriate conditions to achieve coexpression, and this was achieved for all combinations except for the BTB subfamily and Rnd3, where small G protein expression could not be achieved in the presence of RhoGDI-1 or RhoGDI-2. Additionally, the Miro family was successfully coexpressed with the RhoGDIs with the exception that Miro2 could not be coexpressed with RhoGDI-2 (Figure S1). The remaining 6 atypical Rho family GTPases and the successful Miro combinations were individually coexpressed with RhoGDI-1 or RhoGDI-2 and tested for binding as above. RalB was included as a negative control and one of the classical Rho GTPases as a positive control. No interactions were identified between either RhoGDI-1 or RhoGDI-2 and any of the atypical Rho GTPases or Miros that were tested (Figures 4 and 5).

### RhoGDI-3 Interactions with the Rho-Family GTPases and Miros

The final member of the RhoGDI family, RhoGDI-3, is widely expressed but at particularly high levels in the brain, lung, kidney, and testis.^34^ Although less well studied than the other two members of the family, some RhoGDI-3 interacting Rho family G proteins were identified in a yeast two hybrid screen, where mouse RhoGDI-3 was shown to interact with RhoB and RhoG but not with RhoA, RhoC, or Rac1.^34^ An alternative study, using purified proteins, identified interactions between human RhoGDI-3 and both RhoA and Cdc42 but not with Rac1 or Rac2.^35^

Here, FLAG-tagged RhoGDI-3 was coexpressed with V5-tagged versions of all 20 Rho family GTPases, RhoBTB, and the Miro proteins. Trials were carried out to determine the appropriate conditions to achieve coexpression and this was achieved for all combinations except for Rnd1, Rnd2, and RhoBTB proteins, where expression could not be achieved in the presence of RhoGDI-3 (Figure S2). The remaining 16 Rho family and Miro GTPases were taken forward. These were individually coexpressed with RhoGDI-3; lysates were then incubated with beads cross-linked to an anti-FLAG antibody and the precipitated proteins analyzed by Western blotting. RalB was included as a negative control.

**Figure 6:**
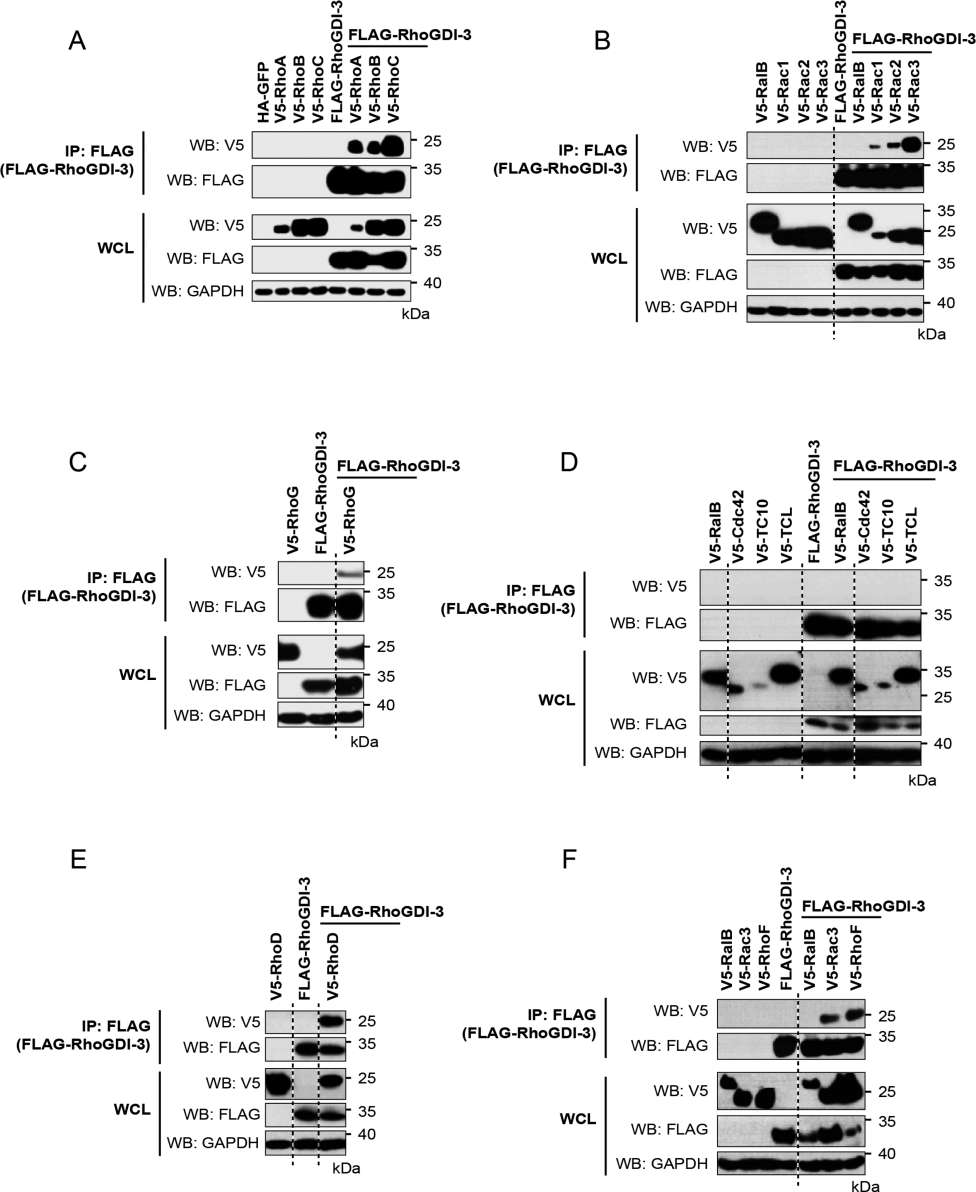
Interaction between RhoGDI-3 and the classical Rho GTPases. V5-tagged constructs of the 12 classical Rho GTPases were expressed alone and with FLAG-tagged RhoGDI-3. Expression of the recombinant proteins in whole cell lysate (WCL) is shown in the bottom panels. RhoGDI-3 was immunoprecipitated from cell lysates using anti-FLAG antibody cross-linked to magnetic beads, and the coimmunoprecipitation of the Rho GTPases was assessed using an anti-V5 antibody (top panels). Results are representative of at least three independent experiments. (A) RhoA, RhoB, and RhoC; (B) Rac1, Rac2, and Rac3; (C) RhoG; (D) Cdc42, TC10/RhoQ, and TCL/RhoJ; (E) RhoD; and (F) RhoF.

**Figure 7:**
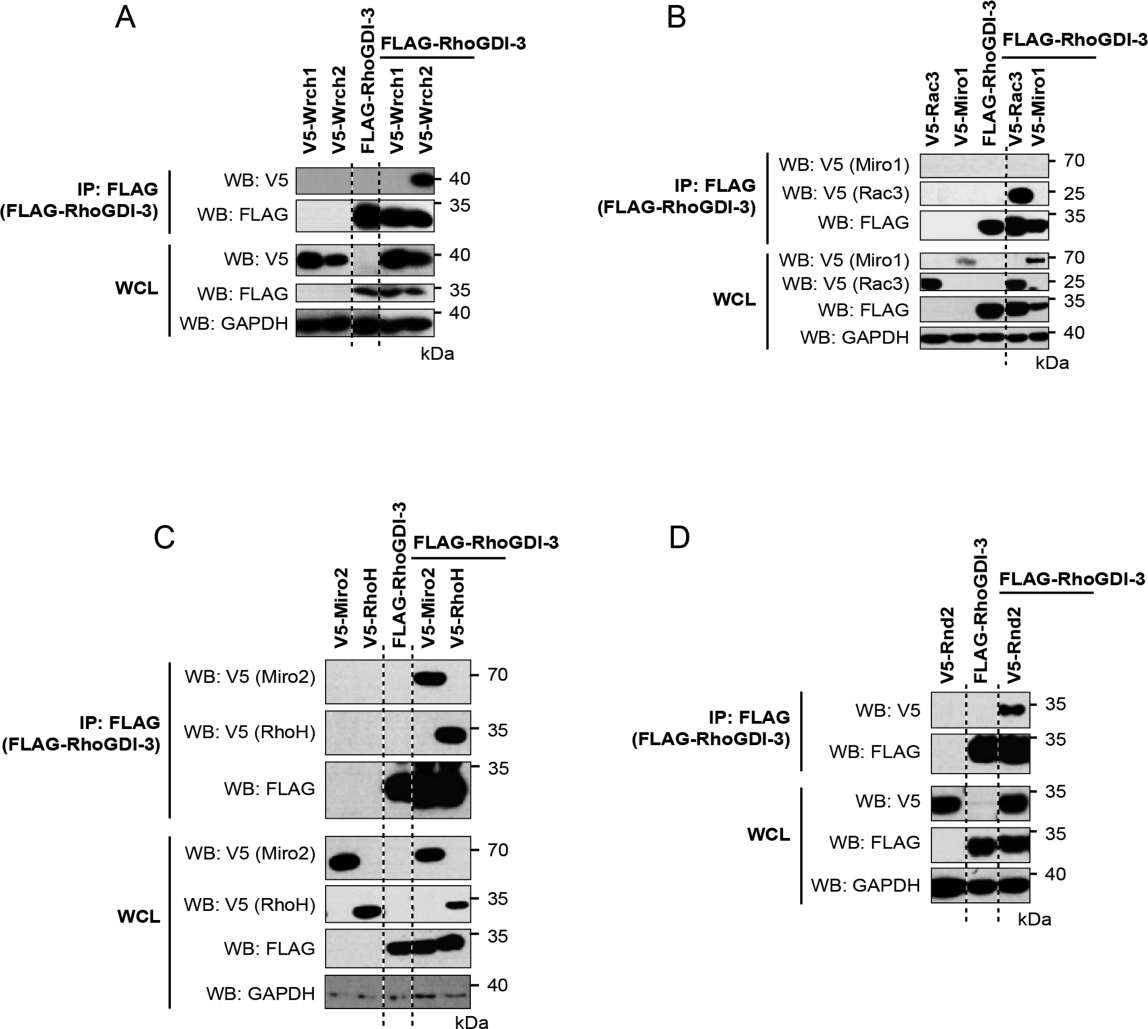
Interaction between RhoGDI-3 and the atypical Rho GTPases. V5-tagged constructs of the 11 atypical Rho GTPases were expressed alone and with FLAG-tagged RhoGDI-3. Expression of the recombinant proteins in whole cell lysate (WCL) is shown in the bottom panels. RhoGDI-3 was immunoprecipitated from cell lysates using anti-FLAG antibody cross-linked to magnetic beads, and the coimmunoprecipitation of the Rho GTPases was assessed using an anti-V5 antibody (top panels). Results are representative of at least three independent experiments. (A) Wrch1/RhoU and Wrch2/RhoV; (B) Miro1; (C) Miro2 and RhoH; and (D) Rnd2.

RhoGDI-3 was found to interact with 12 of the 16 members of Rho family G proteins that could be tested and with one of the Miro proteins. The interactions with RhoA (Figure 6A) and RhoG (Figure 6C) that had been observed previously were confirmed, along with 10 novel interactions: RhoC (Figure 6A); Rac1, Rac2, and Rac3 (Figure 6B); RhoD (Figure 6E); RhoF (Figure 6F); Wrch2/RhoV (Figure 7A); RhoH (Figure 7C); Rnd2 (Figure 7D); and Miro2 (Figure 7C). We could not identify an interaction between RhoGDI-3 and Cdc42 (Figure 6D) in contrast to earlier studies.^35^ Furthermore, no interactions were observed between RhoGDI-3 and the other members of the Cdc42 subfamily, TC10/RhoQ and TCL/RhoJ (Figure 6D), nor with Wrch1/RhoU (Figure 7A) or Miro1 (Figure 7B).

The interactions identified in this screen are summarized in Table 1; green boxes indicate interactions observed in previous studies and confirmed in this work, while yellow boxes highlight the novel interactions identified in this screen.

**Table 1:**
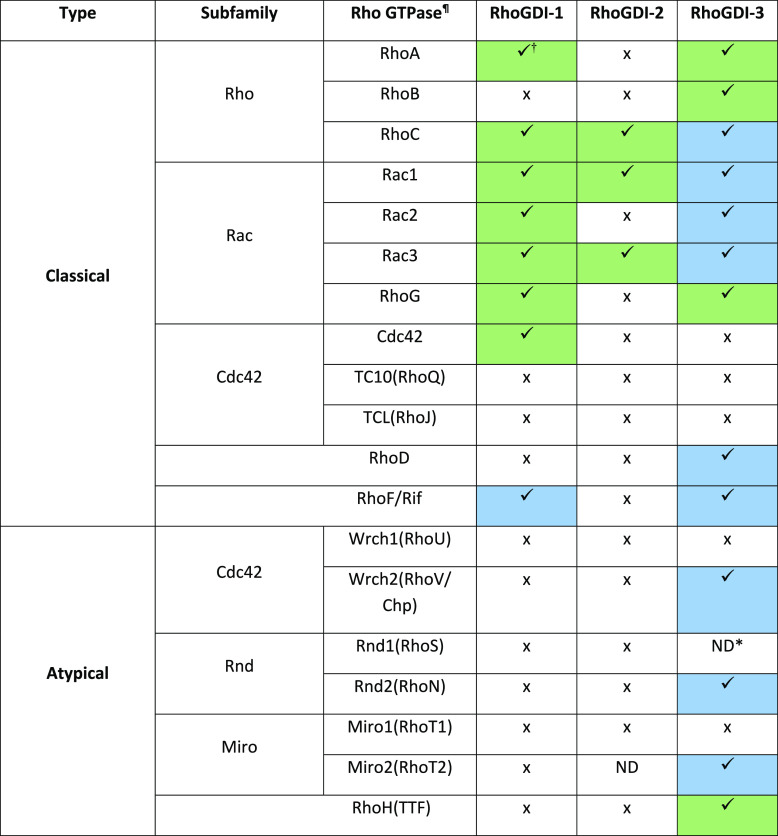
RhoGDI Binding Partners^a^

a Green boxes denote an interaction identified in previous studies and confirmed in this work. Blue boxes denote novel interactions identified in this screen. ¶: Alternative names are shown in brackets. †: Due to the differences in expression levels of the small G proteins, interactions are only described and analyzed qualitatively. *: ND, interaction not studied due to coexpression issues.

### Interactions with Endogenous Proteins

**Figure 8:**
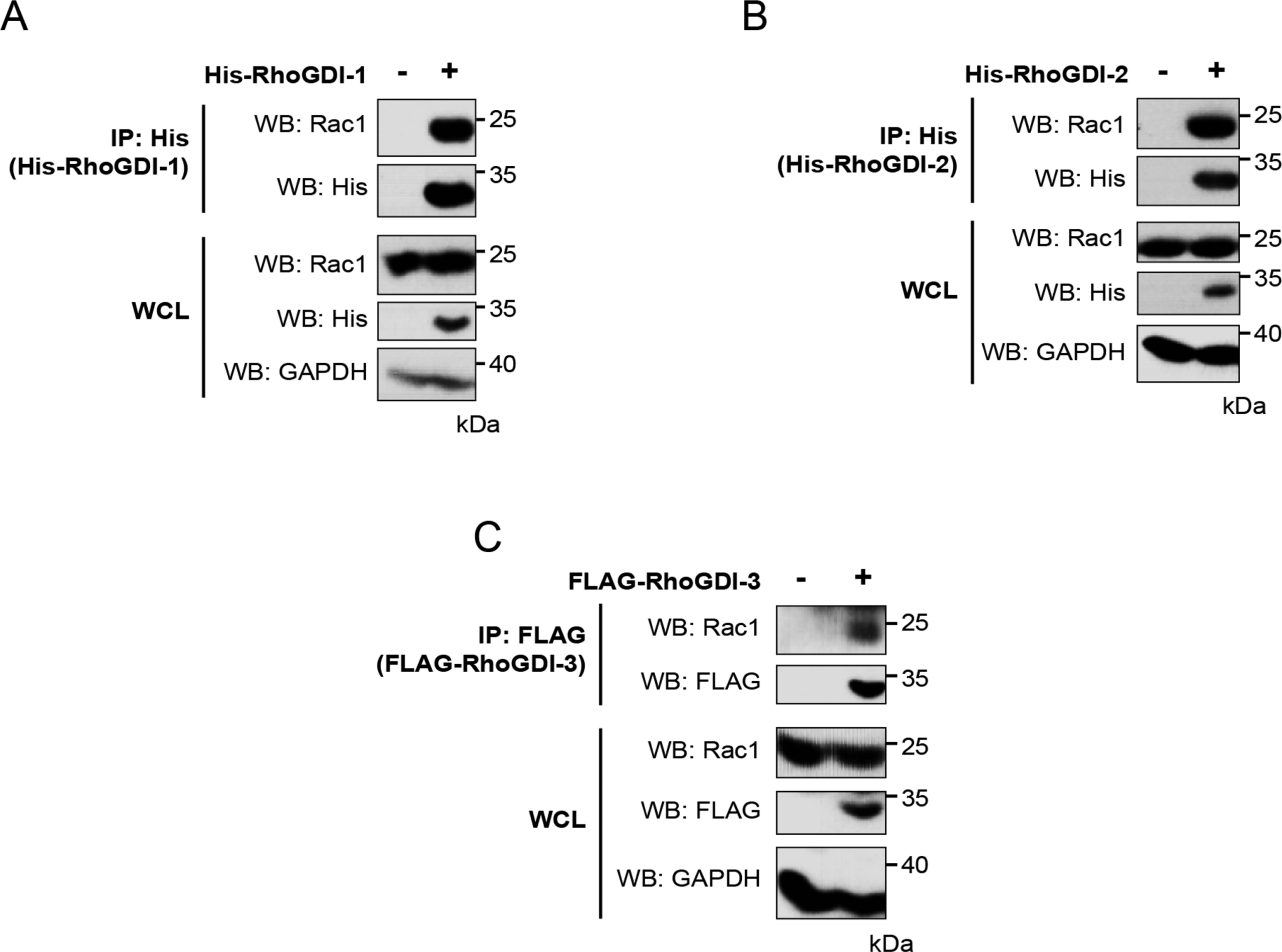
Interaction of exogenous RhoGDIs with endogenous Rac1. FLAG- or His-tagged RhoGDIs were exogenously transfected in HEK293T cells. Expression of the recombinant proteins in whole cell lysate (WCL) is shown in the bottom panels along with the level of endogenous Rac1. RhoGDIs were precipitated from cell lysates using nickle-coated magnetic beads or immunoprecipitated using anti-FLAG antibody cross-linked to magnetic beads. The coimmunoprecipitation of endogenous Rac1 was determine using an anti-Rac1 antibody (top panels). Results are representative of at least three independent experiments. (A) RhoGDI-1, (B) RhoGDI-2, and (C) RhoGDI-3.

All the work presented thus far relied on exogenous expression of all proteins. We wanted to investigate the interactions between endogenous proteins in parallel. However, we encountered problems in studying endogenous RhoGDIs since the commercially available antibodies are not specific, especially those for RhoGDI-3 (Figure S3). Therefore, although we found one antibody specific for RhoGDI-1, as most of the targets had already been identified for this GDI, we chose an alternative strategy and validated one example interaction using exogenously expressed RhoGDIs and endogenous Rac1, which interacted with all three RhoGDIs when coexpressed. We expressed His–RhoGDI-1, His–RhoGDI-2, and FLAG–RhoGDI-3 in HEK293T cells, immunoprecipitated as previously described, and analyzed the samples for the presence of endogenous Rac1 using an anti-Rac1 antibody by Western blotting. All three exogenously expressed versions of the RhoGDI proteins interacted with endogenous Rac1 (Figure 8). Thus, for Rac1 at least, the interactions with exogenously expressed protein mimic those of the endogenous protein.

### RhoGDI-3 Negatively Regulates RhoA, RhoB, RhoC, Rac1, RhoH, and Wrch2/RhoV GTP Levels

**Figure 9:**
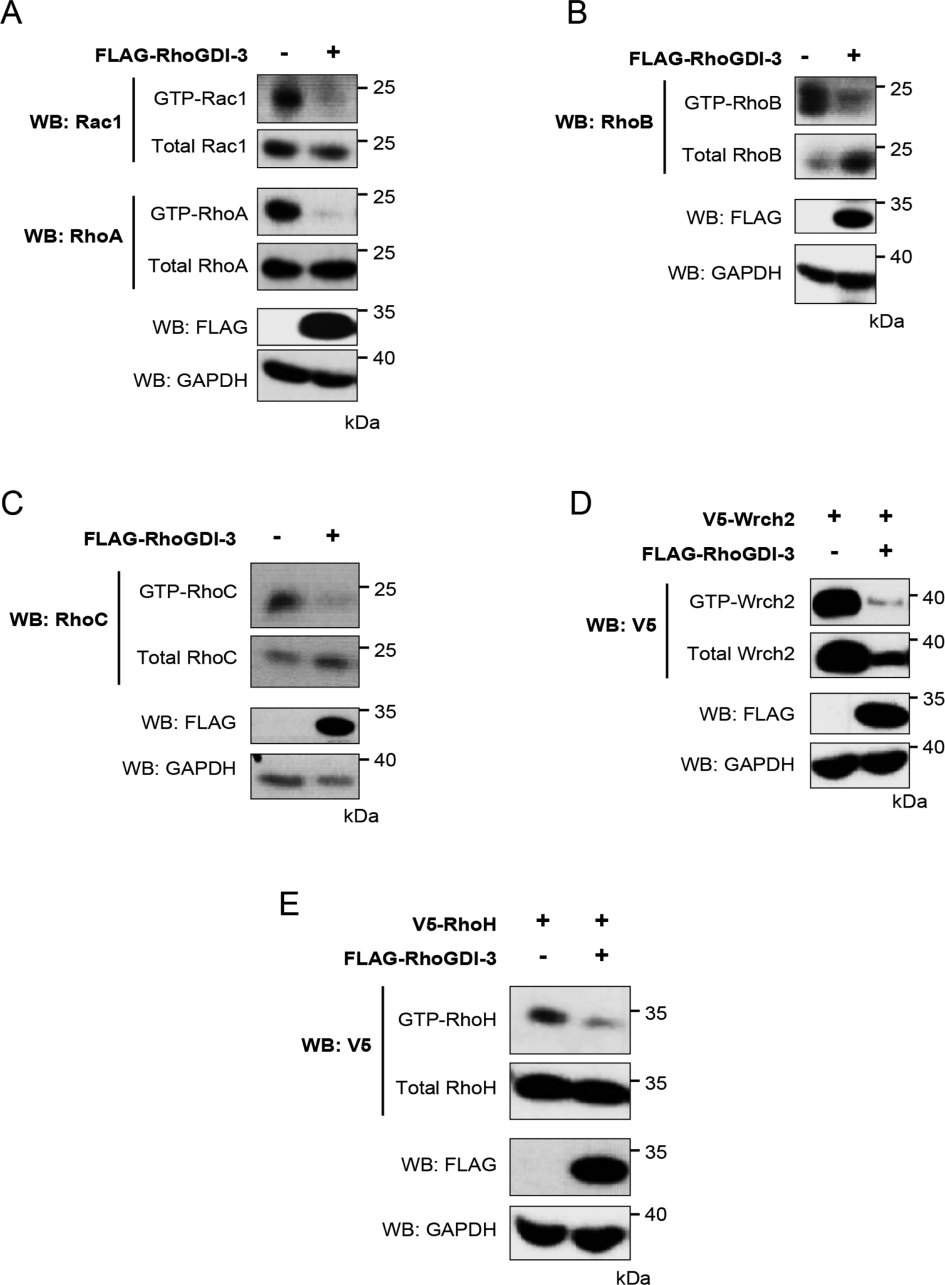
RhoGDI-3 decreases GTP-bound levels of its interacting partners. Levels of GTP-bound Rac1, RhoA, RhoB, RhoC, Wrch2/RhoV, and RhoH were determined in pull-down assays using GST fusion constructs of their effector proteins PAK1 and Rhotekin, in the presence and absence of RhoGDI-3. The Rho GTPases were expressed with and without RhoGDI-3 in HEK293T cells. Expression of the recombinant proteins in whole cell lysate (WCL) is shown in the bottom panels. The GTP-bound Rho GTPases-effector complexes were then precipitated with glutathione-sepharose beads and the levels of GTP-bound Rho GTPases determined by Western blotting with α-Rac1 for GTP-Rac1 or α-V5 for GTP-RhoH and GTP-Wrch2/RhoV (top panels). Results are representative of at least three independent experiments. (A) Rac1 and RhoA; (B) RhoB; (C) RhoC; (D) Wrch2/RhoV; and (E) RhoH.

Our results define the binding profile for the RhoGDI proteins. For RhoGDI-3, we have identified a number of new interaction partners and therefore potential targets for its GDI activity. To determine whether the new binding partners for RhoGDI-3 were also substrates, we took a subset of these putative targets forward for further investigation. We selected RhoA, RhoB, RhoC, and Rac1 as examples of the classical Rho GTPases. We have identified Rac1 as a binding partner for RhoGDI-3 in contrast to previous studies.^34,35^ We also investigated RhoH and Wrch2/RhoV as these are examples of atypical Rho GTPases, which have not been previously identified as RhoGDI-3 partners. To determine the activation status of these small GTPases, we performed effector pull-down assays for all six selected G proteins either alone or when coexpressed with FLAG–RhoGDI-3, to determine the levels of GTP-bound G protein in each case. The data in Figure 9 show significantly decreased levels of the GTP-bound species for all six of these interacting Rho GTPases, suggesting that RhoGDI-3 behaves as a conventional GDI toward its targets.

It is also evident from the data in Figure 9 that RhoGDI-3 affects overall protein levels of its target GTPases and that this effect is target dependent. Levels of RhoB (Figure 9B) and, to a lesser extent, RhoC (Figure 9C) increase in the presence of RhoGDI-3. Levels of RhoA (Figure 9A), Rac1 (Figure 9A), and RhoH (Figure 9E) remain unchanged. However, levels of Wrch2/RhoV decrease significantly (Figure 9D). These data suggest that the protective activity of RhoGDI-3 is target dependent.

## Discussion

Rho family GTPase activation is tightly controlled by a triumvirate of regulatory proteins of which the RhoGDIs are the least well studied. However, aberrant activity of RhoGDIs, through either changes to their cellular levels or modification of their ability to bind to target proteins, has been found to be associated with several disease states, for example, cancer. It is likely that when changes to the RhoGDIs lead to disease, the latter is the consequence of disturbances in the equilibrium of the Rho GTPases themselves. For instance, the interaction of RhoGDI-1 with EphrinB1 has been shown to stimulate RhoA displacement from the RhoA–RhoGDI-1 complex leading to RhoA activation, which promoted breast cancer cell migration.^36^ Furthermore, the RhoGDI-1 interaction with 14–3–3τ has also been shown to support cell migration and invasion in breast cancer by disturbing RhoGDI-1 association with its targets, RhoA, Rac1 and Cdc42.^37^ These data suggest that more detailed knowledge of the RhoGDI function and target proteins could help in the search for new therapeutic avenues in cancer. The RhoGDIs may well also represent an important class of druggable therapeutic targets within small G protein regulated signaling cascades. Modulating membrane localization of small G proteins has had a high profile since farnesyl transferase inhibitors were trialled to suppress Ras activity in cancers.^38^ Although these inhibitors never progressed to clinical utility, renewed interest in preventing membrane localization has re-emerged more recently with the discovery that PDEδ acts as a GDI-like molecule for Ras^39^ and the identification of small molecule inhibitors of PDEδ with biological activity.^40^

**Figure 10:**
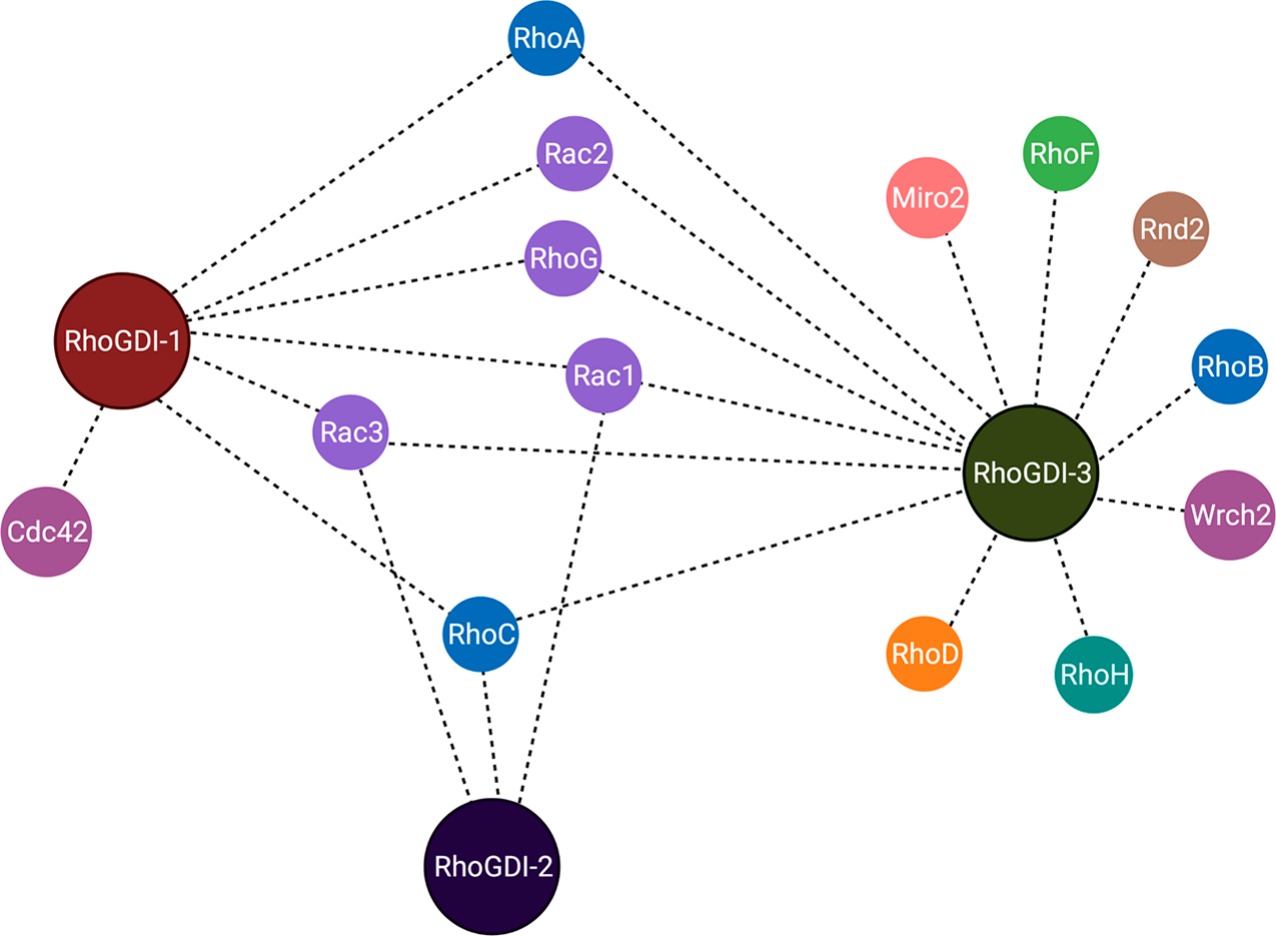
RhoGDI interactome.

To date, relatively few Rho family targets have been identified for the RhoGDIs, and these have been found in a sporadic manner with no systematic survey of the RhoGDI targets undertaken. Validated targets mainly come from the typical or classical small Rho GTPase subfamilies such as RhoA, Rac1, and Cdc42. Almost nothing is known about RhoGDI interactions with the atypical small Rho GTPases. The full binding profile of the RhoGDI proteins is crucial to examine, as the RhoGDIs represent a convergence point for Rho family signaling. The limited availability of the RhoGDIs with respect to their targets defines the relative balance of Rho family GTPase levels in the cell, as well as their activation levels and correct subcellular localization. Although our data are qualitative and do not give any estimates of binding affinity and therefore potential competition between Rho GTPases, they do allow us to analyze the binding complexes in terms of specificity and factors driving complex formation. Our binding data are summarized as an interactome in Figure 10.

### RhoGDI-1

Here, we have found that RhoGDI-1 forms complexes with RhoA, RhoC, Rac1, Rac2, Rac3, RhoG, Cdc42, and RhoF. RhoF is a newly identified target for RhoGDI-1, and this is consistent with its similarity to Rac and Rho subfamily members (∼50%, see Table S1). Although these small G proteins have multiple cellular functions, all of the targets of RhoGDI-1 have roles in regulating the actin cytoskeleton.

The structure of RhoGDI-1 in complex with isoprenylated Cdc42 identified key contacts to residues in Cdc42 in switches I (Thr35 and Val36) and II (Ala59, Tyr64, Arg66, Leu67, and Leu70), in the polybasic region (Arg186 and Arg187) and Glu95, Glu100, His 103, and 104 in helix α3. All of the targets we have identified for RhoGDI-1 show good but not perfect conservation of these side chains (Figure 1B, Table 2), allowing the specificity determinants for RhoGDI-1 to be assessed.

**Table 2:** Summary of Contacts between Cdc42 and RhoGDI-1 and Their Conservation in Rho Proteins

Rho GTPase	switch I contacts	switch II contacts	helix α3 contacts	PBR contacts^a^	GDI-1 binding	conservation/explanation for lack of binding
Cdc42	Thr^35^, Val^36^	Ala^59^, Tyr^64^, Arg^66^, Leu^67^, Leu^70^	Glu^95^, Glu^100^, His^103^, His^104^	Arg^186^, Arg^187^	yes	
RhoA	Thr, Val	Ala, Tyr, Arg, Leu, Leu	Glu, Glu, His, Phe	Lys, Lys, Lys	yes	all
RhoB	Thr, Val	Ala, Tyr, Arg, Leu, Leu	Glu, Glu, His, Phe	None	no	all, no PBR, palmitoylated
RhoC	Thr, Val	Ala, Tyr, Arg, Leu, Leu	Glu, Glu, His, Phe	Lys, Arg, Arg, Arg	yes	all
Rac1	Thr, Val	Ala, Tyr, Arg, Leu, Leu	Ala, Glu, His, His	Lys, Arg, Lys	yes	Glu^95^→Ala
Rac2	Thr, Val	Ala, Tyr, Arg, Leu, Leu	Ala, Glu, His, His	Lys, Lys	yes	Glu^95^→Ala
Rac3	Thr, Val	Ala, Tyr, Arg, Leu, Leu	Ala, Glu, His, His	Lys, Lys	yes	Glu^95^→Ala
RhoG	Thr, Val	Ala, Tyr, Arg, Leu, Leu	His, Glu, His, His	Arg	yes	Glu^95^→His
TC10/RhoQ	Thr, Val	Ala, Tyr, Arg, Leu, Leu	Glu, Glu, Glu, Tyr	Lys, Lys, Arg	no	His^103^→Glu
TCL/RhoJ	Thr, Val	Ala, Tyr, Gln, Leu, Leu	Glu, Glu, Asp, Cys	Lys, Lys, Arg	no	Arg^66^→Gln, His^103^→Asp
RhoD	Thr, Val	Ala, Tyr, Arg, Leu, Leu	Asn, Glu, His, Phe	Arg, Arg	no	Glu^95^→Asn, farnesylated
RhoF	Ser, Val	Ala, Tyr, Arg, Leu, Leu	Ile, Glu, His, Phe	Arg, Lys, Lys, Arg, Arg	yes	Glu^95^→Ile
Wrch1/RhoU	Thr, Ala	Ala, Phe, Lys, Leu, Leu	Glu, Glu, Cys, His	Lys, Arg, Lys, Lys, Lys	no	Val^36^→Ala, Tyr^64^→Phe, His^103^→Cys
Wrch2/RhoV	Thr, Ala	Ala, Phe, Arg, Leu, Leu	Glu, Glu, Thr, His	Lys, Lys, Lys, Arg, Arg	no	Val^36^→Ala, Tyr^64^→Phe, His^103^→Thr
Rnd1	Thr, Val	Ser, Tyr, Asn, Val, Leu	Lys, Glu, Asp, Tyr	Lys, Arg, Lys, Arg	no	Arg^66^→Asn, Glu^95^→Lys, His^103^→Asp
Rnd2	Thr, Val	Ser, Tyr, Asn, Val, Leu	Lys, Glu, Glu, Phe	Arg, Arg, Arg	no	Arg^66^→Asn, Glu^95^→Lys, His^103^→Glu
RhoH	Thr, Val	Ala, Phe, Ser, Ile, Leu	Asn, Glu, Ser, Asn	Arg, Arg, Arg	no	Tyr^64^→Phe, Arg^66^→Ser, Glu^95^→Asn, His^103^→Ser

a Given the dynamic nature of the C-terminus, residues further from the G domain are listed but not those that are closer to the final α-helix, which are likely to be unavailable for binding.

The Ala–Tyr–Arg triplet in switch II is conserved in all the targets, and the importance of Arg66 for the Cdc42–RhoGDI-1 interaction has been shown previously by mutagenesis.^41^ His103 is an important contact site between Rac1 and RhoGDI-1^42^ and forms a salt bridge with Asp184 in RhoGDI-1 as well as stacking against the ring of Tyr27^RhoGDI-1^, with Met145 completing the contacts to the His (Figure 10). His103 is conserved in all of the RhoGDI-1 targets, providing an explanation for the lack of binding of the Cdc42 subfamily members, TC10/RhoQ, TCL/RhoJ, and the Wrch proteins. The remainder of the Rho family proteins that do not bind RhoGDI-1 diverge from the Cdc42 contact sites for RhoGDI-1 at multiple positions (Table 2).

**Figure 11:**
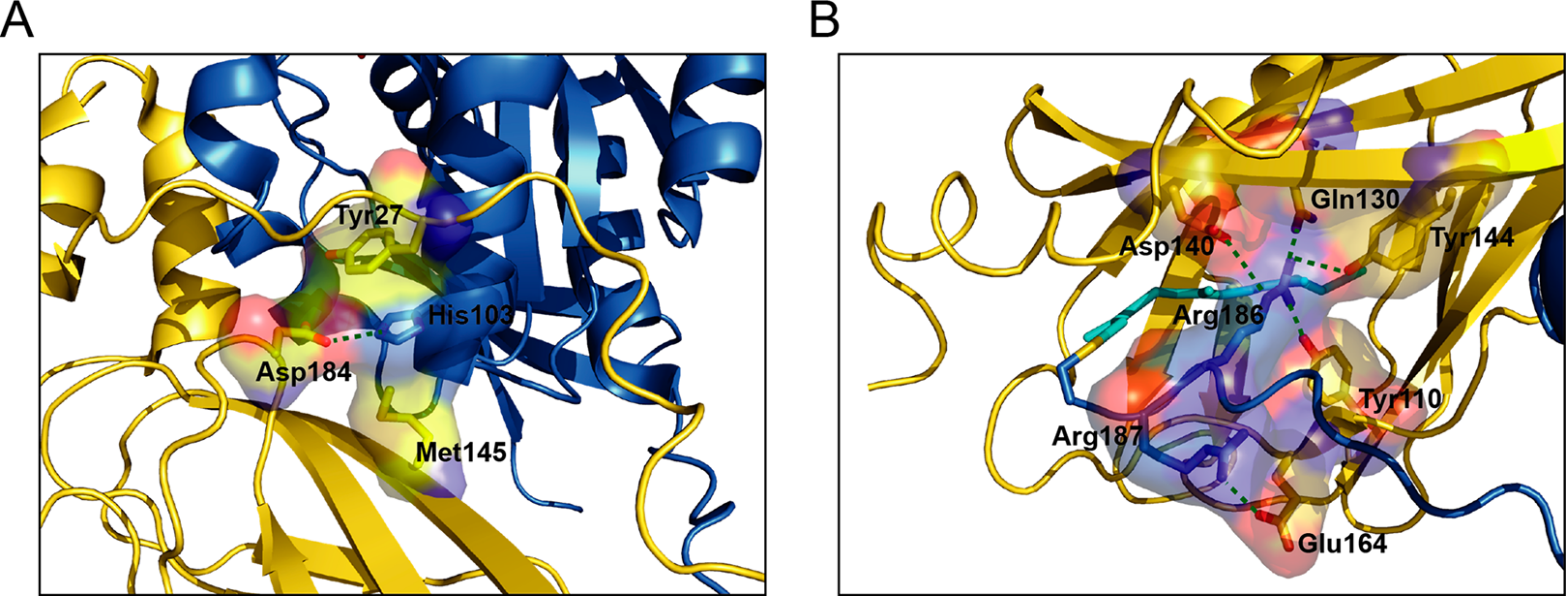
Potential discriminatory interactions of RhoGDI-1. Interactions involving Cdc42 (A) His103 and (B) Arg186–Arg186 may be involved in the selecting of specific Rho family protein binding. Cdc42 is shown in blue, and RhoGDI-1 is shown in gold. The nucleotide is shown in a stick representation with carbons in green, oxygens in red, nitrogens in blue, and phosphoruses in orange. The Mg^2+^ ion is shown as a pink sphere. The geranylgeranyl group is shown in cyan as a stick representation. Residues involved in interactions are labeled and salt bridges/hydrogen bonds are shown as green dashed lines.

Residues Arg186 and Arg187 in the polybasic region of Cdc42 make contacts with a polar patch on RhoGDI-1, which has been postulated to contribute to the membrane release mechanism of the GDIs.^43^ Arg186 of Cdc42 extends the lipid binding pocket of RhoGDI-1, capping the geranylgeranyl moiety sequestered by RhoGDI-1, and makes hydrogen bonds with GDI residues Tyr110, Gln130, Asp140, and Tyr144. Arg187 is more exposed but forms a salt bridge with Glu164 (Figure 11). The double Arg at the C-terminus of Cdc42 is in the hypervariable region, which is rich in basic residues and lies between the end of the G domain and the C-terminal prenylated Cys. Despite being involved in interactions between Cdc42 and RhoGDI-1, the C-terminal hypervariable regions of the Rho proteins remain flexible within the complex. This, along with the number of basic residues, means that a certain amount of promiscuity of binding will be possible, so that the precise nature and positioning of the basic residues are not important. Hence, it was shown that when RhoA binds to RhoGDI-1, three Lys residues in the hypervariable region were in a position to form contacts with the same site on RhoGDI-1.^44^ In the Rac1–RhoGDI-1 complex, Lys186/188 form contacts with this patch of RhoGDI-1, although the details of the hydrogen bonds/salt bridges formed are not the same. This flexibility in the binding site means that Rac2 and Rac3 can still bind to RhoGDI-1, even though their dilysine sequences do not precisely align with that of Cdc42 (Figure 1B).

RhoB does not bind to RhoGDI-1 because, although it has a Lys–Arg sequence, it is adjacent to the end of helix α5 and would not be close enough to the GDI. Furthermore, RhoB is palmitoylated as well as prenylated, and palmitoylation directly adjacent to the prenyl group has been shown to inhibit the interaction with RhoGDI-1.^45^

We did not identify RhoD as a target for RhoGDI-1. This is hard to reconcile as RhoD satisfies all of the contacts described above (Table 2). RhoD is, however, thought to be farnesylated,^6^ and all the targets we see for RhoGDI-1 are geranylgeranylated, suggesting that contacts with the longer prenyl group are important for RhoGDI-1 complexes.

### RhoGDI-2

Our data indicate that RhoGDI-2 has a more limited target profile, which includes only RhoC, Rac1, and Rac3. Early studies suggested that RhoGDI-2 had a restricted expression profile and was confined to hematopoietic cells; however, more recent data show expression in a wider range of tissues and cancer cell types.^46^ Rac1 and Rac3 are expressed well in hematopoietic cells but interestingly RhoC is not.^47^ However, RhoGDI-2 expression is seen in other cell types where RhoC is also found,^47^ and RhoC has also been identified as a substrate for RhoGDI-2 in bladder cancer cells.^33^ Previous studies identified Cdc42^48^ and RhoA^33^ as RhoGDI-2 targets. However, these were not observed in the system used in this study. We cannot rule out the possibility that tissue- or cell-specific modifications to either the GTPases or the GDI proteins are involved in some interactions, and these would not necessarily be present in our system or may differ between alternative experimental systems. RhoGDI-2 has also been reported to make weaker complexes with its Rho GTPase targets. For instance, RhoGDI-2 was shown to bind to Cdc42 with a 10- to 20-fold lower affinity than RhoGDI-1.^49,50^ One possible explanation for the decreased binding affinity of RhoGDI-2 for its targets has been suggested to be the presence of Asn174, in a position analogous to Ile177^RhoGDI-1^ (Figure 1C). This polar residue could potentially disrupt the hydrophobic environment of the RhoGDI Ig domain and thus reduce affinity for the isoprenyl group of the GTPase targets. An interaction between RhoGDI-2 and Rac2, however, was predicted prior to this screen, as a crystal structure of the RhoGDI-2-Rac2 complex has been solved.^32^ However, this structure showed some curious features. Although mass spectrometric analysis of the purified Rac2–RhoGDI-2 complex was consistent with the presence of full-length isoprenylated Rac2, no electron density for the C-terminal residues or the isoprene were visible in the data, so the contribution of Asn174 remains unknown. Otherwise, many of the residues of Rac2 in contact with RhoGDI-2 were similar to those on Cdc42 in contact with RhoGDI-1, with Arg66 playing a key anchor role again. Other differences between RhoGDI-1 and RhoGDI-2 lie in residues 142–144 (Thr–Asp–Tyr in RhoGDI-1 and Ala–Thr–Phe in RhoGDI-2); these are located in the lipid-binding domain of the RhoGDIs, so their function in RhoGDI-2 also remains elusive currently. Nonetheless, no interaction was observed between Rac2 and RhoGDI-2 in this study, indicating that these proteins might not interact in a cellular environment. However, due to potential low affinity between RhoGDI-2 and its binding targets, it cannot be ruled out that some RhoGDI-2 targets may be unaccounted for in this study.

### RhoGDI-3

The least studied RhoGDI, RhoGDI-3, has been shown here to engage with the widest range of target GTPases of all the GDIs, binding to all the typical members of the Rho-family GTPases except Cdc42, TC10/RhoQ, and TCL/RhoJ. It was also found to associate with several atypical Rho-family GTPases such as RhoD, RhoH, Wrch2/RhoV, and Rnd2, and also with Miro2.

RhoGDI-3 is very similar to RhoGDI-1 in all positions which represent contact sites with Cdc42 (Figure 1C), explaining why it can complex with most RhoGDI-1 targets. Interestingly, however, we did not see an interaction between RhoGDI-3 and Cdc42. RhoGDI-3 has a histidine at position 130, which is a glutamine in RhoGDI-1. Gln130^RhoGDI-1^ contacts Arg186 in Cdc42 and also lines the lipid binding pocket. Replacement of Gln130 in RhoGDI-3 with histidine could decrease binding affinity to Cdc42 but would be tolerated by other RhoGDI-3 targets, suggesting that the details of lipid binding by RhoGDI-3 may be slightly different to those of RhoGDI-1.

Most notably, RhoGDI-3 binds to a series of Rho GTPases that do not interact with the other two GDIs, including RhoB, Rac2, Wrch2/RhoV, Rnd2, Miro2, RhoD, and RhoH. The interaction between RhoGDI-3 and the atypical, GTPase defective Rho family GTPases such as RhoH, Wrch2/RhoV, and Rnd2 was unexpected as these Rho GTPases have been shown to be constitutively active due to their high intrinsic GDP dissociation rate.^51,52^ Additionally, it has been reported that none of the three RhoGDIs prevented Wrch2/RhoV membrane association, suggesting that localization of Wrch2/RhoV at least is not regulated by the RhoGDIs.^53^ However, RhoGDI-1 has been observed to accommodate both the GTP- and GDP-bound forms of Rac1, RhoA,^54^ and Cdc42.^50^ This has been supported by structural studies that show that the main interface between RhoGDI-1 and Cdc42 is not affected by the nucleotide state of Cdc42,^55^ suggesting that GTP-bound GTPases may also be targeted by RhoGDIs. There is also a possibility that RhoGDI-3 regulates the activity of these unusual Rho family GTPases via an adaptor protein, 14–3–3, consistent with previous studies that showed that 14–3–3ß negatively regulates Rnd activation^11^ and also binds to RhoGDI-1.^37^ However, our data show that RhoGDI-3 not only binds to these atypical Rho-family GTPases but also, in the cases of RhoH and Wrch2/RhoV at least, decreases the levels of GTP-bound G protein, thus functioning as a conventional GDI toward these targets.

Interestingly, Wrch2/RhoV has been shown to be modified solely by palmitoylation. Previously, palmitoylation has been reported to abrogate the interaction between RhoGDI-1 and RhoA when an extra Cys was introduced adjacent to the isoprenyl site.^41^ Conversely, however, a study by Navarro-Lérida et al. showed that RhoGDI-1 was able to bind to palmitoylated Rac1.^56^ This is easily rationalized as the palmitoylation site on Rac1 is distant from the contacts to the lipid binding pocket and so modification at both sites is compatible with formation of a RhoGDI complex. Since Wrch2/RhoV was only found to interact with RhoGDI-3 and not with the other RhoGDIs, it is also possible that the lipid binding domain of RhoGDI-3 can accommodate palmitoylated Rho GTPases, where the palmitoyl moiety is in an analogous position to the more usual prenyl, suggesting that RhoGDI-3 is potentially involved in targeting these GTPases to specific subcellular compartments. It is also notable that some of the targets of RhoGDI-3 are farnesylated rather than geranygeranylated, suggesting again that the lipid binding domain of RhoGDI-3 may be more flexible in the substrates that it can accommodate. Indeed, the binding profile that we have revealed suggests that RhoGDI-1 and RhoGDI-2 exclusively bind geranylgeranylated proteins while RhoGDI-3 can interact with geranylgeranylated, farnesylated, both prenylated and palmitoylated proteins and, in the case of Wrch2/RhoV, nonprenylated targets. There is also no evidence to suggest that Wrch2/RhoV is truncated at its C-terminus as this occurs after prenylation in small GTPases, suggesting that the hydrophobic Phe–Val doublet would also be available at the C-terminus of Wrch2/RhoV (see below).

The major difference between RhoGDI-3 and the other two GDIs is the presence of an extended N-terminal region in RhoGDI-3 (Figure 1C). The N-terminal half of the RhoGDIs is an intrinsically disordered region, part of which undergoes a disorder–order transition on binding to the switch regions of the Rho protein targets, forming a helix–loop–helix domain that acts to inhibit nucleotide exchange. A portion of RhoGDI-1 at the extreme N-termini remains unstructured within the complex and is missing in several structures or has high temperature factors, indicating that it is still dynamic in the complex. The length of this extreme N-terminal region varies between the three RhoGDIs, being 33 residues in RhoGDI-1, 29 residues in RhoGDI-2, and 54 residues in RhoDGI-3. In all three GDIs, this region contains multiple acidic residues (8, 9, and 9, respectively) which are concentrated in RhoGDI-1 and RhoGDI-2 into an acidic patch central to the region (Figure 1C). These acidic residues in RhoGDI-1 may form contacts with the polybasic region in Cdc42 in the encounter complex and so are not seen in the final complex, being involved in the mechanism of membrane release by competing with the membrane lipids for the polybasic region. The acidic residues in the N-terminal region of RhoGDI-3 are more dispersed but still feature close to the N-terminal helix–loop–helix domain in a position where they could contact the polybasic region of a target.

**Figure 12:**
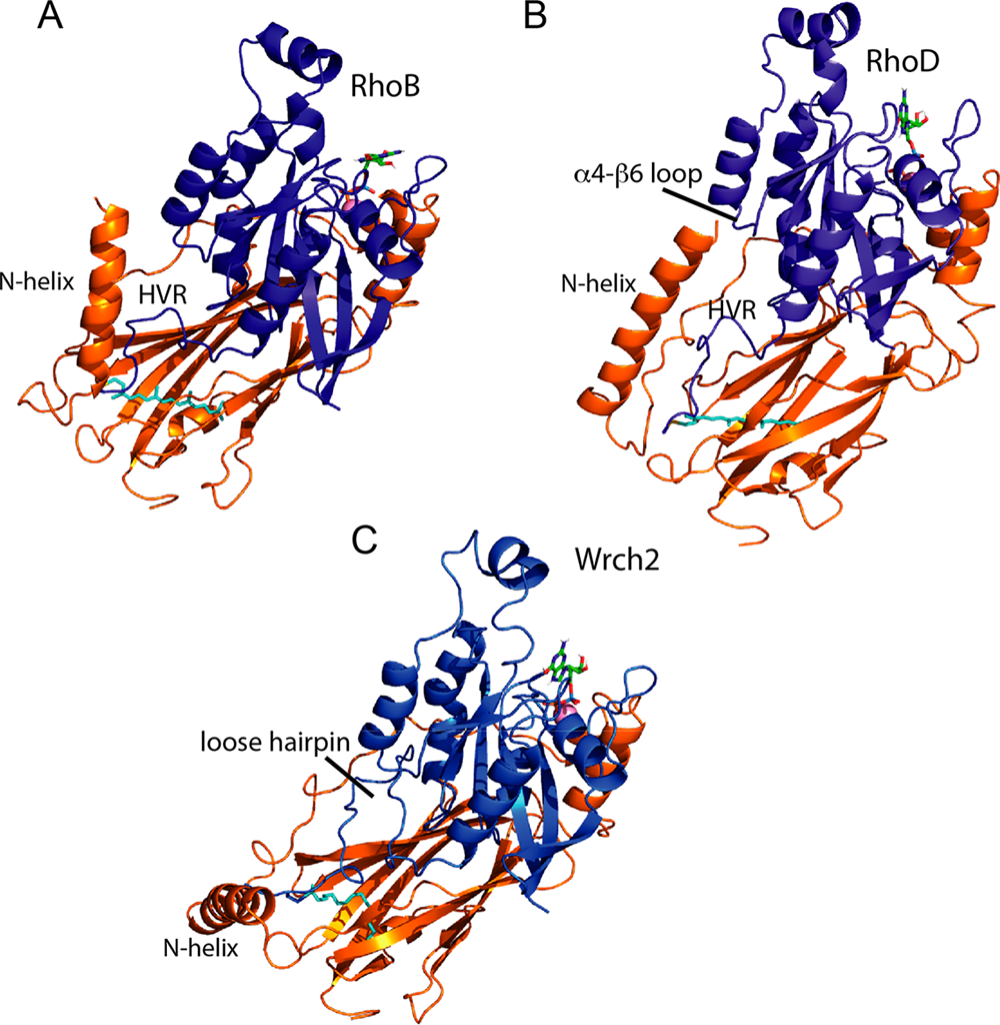
Models of RhoGDI-3 bound to three Rho-family proteins. Models of three representative Rho-family proteins with RhoGDI-3. (A) RhoB–RhoGDI-3, (B) RhoD–RhoGDI-3, and (C) Wrch2–RhoGI-3. In each model, the N-terminal helix of RhoGDI-3 is in a different orientation, making contacts with the hypervariable region (HVR, RhoB) or the α4-β6 loop (RhoD). In the Wrch2/RhoV model, the extended HVR forms a loose hairpin that lies on the RhoGDI-3 Ig domain. The Rho family proteins are shown in blue and RhoGDI-3 is colored orange. GDP is shown in a stick representation with carbons in green, oxygens in red, and nitrogens in blue. The lipid moiety, geranylgeranyl (RhoB and RhoD) or palmitoyl (Wrch2/RhoV), attached to the Rho protein is shown in cyan.

Toward its extreme N-terminus, RhoGDI-3 contains a predicted amphipathic helix which has been demonstrated to play a role in both stabilization of the RhoGDI-3–RhoG complex and also in targeting RhoGDI-3 to the Golgi apparatus.^16^ We speculated that this extended N-terminal region of RhoGDI-3 may play a role in broadening the target profile of RhoGDI-3 and therefore built structural models of RhoGDI-3 in complex with three targets, RhoB, RhoD, and Wrch2/RhoV, based on the Cdc42–RhoGDI-1 and Rac1–RhoGDI-1 structures. These templates were chosen because they have the most structural information about the N-terminus of the GDI. Wrch2/RhoV was modeled with a palmitoyl on the C-terminal Cys, overlaid on the C-terminal Cys of Cdc42 so that it occupied the isoprenyl binding pocket of the GDI. The isoprenyl binding pocket has some flexibility in its lipid binding, since it can accommodate farnesyl as well as geranylgeranyl.^57^ Preliminary models of all three complexes were refined using Haddock2.4^27^ with full flexibility for the N-terminus of RhoGDI-3 and the C-terminus of the G protein. In all three models, the N-terminal helix of the RhoGDI-3 turns back toward the main body of the GDI (Figure 12). This is due to a Gly–Gly–Pro–Pro sequence, which causes a kink in the protein backbone, between the N-terminal helix and the start of the region homologous to the other GDIs. In the RhoB and RhoD complexes, the helix lies on top of the Rho protein C-terminal hypervariable region, suggesting that it could make contacts with residues there. The RhoGDI-3 helix is rich in hydrophobic amino acids and it is possible that it contacts hydrophobic side chains in the hypervariable region, particularly in RhoD, which has two Phe and a Trp residue here (Figure 1B). The RhoGDI-3 helix also extends toward the G-domain in both models, making contact with the loop between helix α4 and strand β6. This short loop is divergent between the different G proteins, varying in both charge and sequence, and it may therefore allow some discrimination between proteins that bind to RhoGDI-3 and those that do not. For example, Cdc42 and Wrch1/RhoU, neither of which bind to RhoGDI-3, both have a Lys at position 150 (Cdc42 numbering) although it is unlikely that charge alone determines binding since RhoF and RhoH both have Arg at this position. In the Wrch2/RhoV–RhoGDI-3 model, the N-terminal RhoGDI-3 helix is prevented from contacting the G domain and instead stands away from both components of the complex. This is due to differences in the C-terminal region of Wrch2/RhoV. As Wrch2/RhoV is not isoprenylated, it is presumably not subject to proteolytic removal of the residues C-terminal to the modified Cys, and so there are an extra two residues at the C-terminus that prevent the Wrch2/RhoV helix from fully folding back onto the C-terminus of Wrch2/RhoV. These two hydrophobic residues are in close proximity to the hydrophobic residues of the RhoGDI-3 amphipathic helix and so may well further stabilize the complex. In addition, the Wrch2/RhoV C-terminal hypervariable region is significantly longer than those of RhoB and RhoD. In all the top models produced by MODELER,^25^ this extra sequence forms a loose hairpin that lies on top of the β-sandwich of the GDI Ig domain and is not available to contact the N-terminal helix of the RhoGDI-3.

Taken together, the extra binding interfaces provided by the N-terminal helix in RhoGDI-3 may explain the extended target profile we see in this study for RhoGDI-3, particularly for the Rho family proteins with shorter C-terminal hypervariable regions such as RhoB. We predict that the increase in potential contact sites on RhoGDI-3 for targets would allow more variations in each individual contact region and support sufficient affinity across multiple functional complexes identified as seen in this study. The first 25 amino acids of RhoGDI-1 have been shown to play a pivotal role in retaining RhoGDI-1 in the cytoplasm, suggesting that the N-termini of the RhoGDI-1 and RhoGDI-2 are also likely to be important in regulating their subcellular localization and therefore facilitate the binding with Rho GTPases that reside within the same compartments.^58^ In the same study, it was found that RhoGDI-1 also localized to phagosomes, and this required an interaction with Rac1, indicating that the RhoGDIs and Rho GTPases are mutually involved in the signal directing them to specific cell compartments.

As well as the Golgi apparatus, RhoGDI-3 has been found to localize to endomembranes and early endosomes.^16^ Several Rho family GTPases have also been found to localize at these same sites, for example, RhoB,^59^ Rac3,^60^ RhoG,^61^ Wrch2/RhoV,^62^ Rnd2,^63^ and RhoD.^64^ This suggests that RhoGDI-3 might interact with its partners at endomembranes and play a role in regulating their intracellular trafficking. This hypothesis is consistent with our findings that show that RhoGDI-3 interacts with all of the endomembrane-associated small Rho GTPases cited above.

The Miros, mitochondria-associated small GTPases, were originally classified as atypical Rho family members. They possess two G domains: the first resembles Rho family proteins and the second is more similar to Rab family GTPases. They are however quite diverged from Rho sequences and are therefore now considered to be a family of their own: we included them here for completeness. Miro2, but not Miro1, was shown to be a target for RhoGDI-3 although both are structurally similar and localize to the same cell compartment. Nevertheless, these proteins have been shown to be functionally different with the degradation of Miro2 and not Miro1 found to inhibit mitochondrial retrograde trafficking.^65^ Furthermore, Miro2 did not rescue neural respiratory defects due to the loss of Miro1 function in Miro1 knock out mice.^66^ The molecular details of the RhoGDI-3-Miro2 interaction are however unclear as Miro2 does not undergo any lipid modification due to the lack of a CAAX motif and palmitoylated cysteines. These data indicate that RhoGDI-3 may have the capacity to interact with nonlipidated targets, potentially due to the extra contacts formed through its extended N-terminus, and this indicates a new role for RhoGDI-3 in regulating mitochondrial processes through its target, Miro2.

We were unable to fully test all Rnd proteins as RhoGDI partners due to expressions issues, although we identified a RhoGDI-3–Rnd2 interaction suggesting that the Rnd proteins could well be targets for the GDIs.

Interestingly, we found no RhoGDIs which were able to complex with TC10/RhoQ, TCL/RhoJ, Wrch1/RhoU, or Miro1. This would indicate that these particular small G proteins do not require a GDI to support their cellular functions. It is possible that some of these proteins need to turn over quickly and therefore have no need of a chaperone; however, very little information is available for the relative half-lives of the Rho-family proteins. It is also possible that these small GTPases function at only one compartment and therefore reside solely on one membrane type, precluding their need for a chaperone.

## Conclusions

Defining rules for GDI–target engagement so far have been confounded by data suggesting that each individual GDI–G protein complex has unique features, and the consequences of complex formation are context dependent. This is reflected in our own results where little overall consensus is obvious as interactions seem to be governed by multiple different properties of both the G proteins and regions of the GDI. There exists a dynamic balance between members of the Rho GTPases in cells, which is fine-tuned by the RhoGDIs, with the RhoGDIs controlling the crosstalk between the Rho family members. This system is highly sensitive to the relative abundance of all members including the RhoGDIs and their target G proteins and doubtless other regulatory proteins. Our data, especially revealing the broad target range of RhoGDI-3, add to our knowledge of factors governing this complex equilibrium.

## Supporting Information Available

Pairwise identity of the Rho-family and Miro proteins; BTB subfamily, Rnd subfamily, and Miro2 not expressing to detectable levels in the presence of RhoGDIs; coexpression of the BTB subfamily and Rnd subfamily with RhoGDI-3; analysis of RhoGDI antibody specificity (PDF)

## Accession Codes

RhoGDI-1, P52565; RhoGDI-1, P52566; RhoGDI-3, Q99819; RhoA, P61586; RhoB, P62745; RhoC, P08134; Rac1, P63000; Rac2, P15153; Rac3, P60763; RhoG, P84095; CDC42, P60953; TC10 (RhoQ), P17081; TCL (RhoJ), Q9H4E5; Wrch1 (RhoU), Q7L0Q8; Wrch2 (RhoV, Chp), Q96L33; Rnd1 (RhoS), Q92730; Rnd2 (RhoN), P52198; Rnd3 (RhoE), P61587; RhoH (TTF), Q15669; RhoD, O00212; RhoF (Rif), Q9HBH0; Miro1, Q8IXI2; Miro2, Q8IXI1; RhoBTB1, O94844; RhoBTB2, Q9BYZ6; PAK1, Q13153; Rhotekin, Q9BST9

## Abbreviations

GAP: GTPase activating protein
GDI: guanine nucleotide dissociation inhibitor
GDP: guanosine-5′-diphosphate
GEF: guanine nucleotide exchange factor
GTP: guanosine-5′-triphosphate
Ig: immunoglobulin-like
PBD: p21 binding domain
PBR: polybasic region
