# Supplementary material for: A Complete Survey of RhoGDI Targets Reveals Novel Interactions with Atypical Small GTPases
Source: Biochemistry. 2021 Apr 29;60(19):1533–51. doi: 10.1021/acs.biochem.1c00120 (PMC8253491; doi:10.1021/acs.biochem.1c00120)
Supplement: Supplementary file 1 — bi1c00120_si_001.pdf [file bi1c00120_si_001.pdf]

Supporting Information

A complete survey of RhoGDI targets reveals novel interactions with atypical small GTPases

Ana Masara binti Ahmad Mokhtar[†‡], Samrein B. M. Ahmed[†¶], Nicola J. Darling[†§], Matthew Harris [†¥], Helen R. Mott[†*] and Darerca Owen[†*]

Table S1: Pairwise identity of the Rho-family and Miro proteins

Figure S1: The BTB subfamily, Rnd subfamily and Miro2 do not express to detectable levels in the presence of with RhoGDIs.

Figure S2: Co-expression of the BTB subfamily and Rnd subfamily with RhoGDI-3. V5

Figure S3: Analysis of RhoGDI antibody specificity

Table S1: Pairwise identity of the Rho-family and Miro proteins

	RHOA	RHOC	RHOB	RAC1	RAC3	RAC2	RHOG	CDC42	TC10	TCL	WRCH1	WRCH2	RND1	RND2	RND3	RHOD	RHOF	RHOH	BTB1	BTB2	MIRO1A	MIRO2A	MIRO1B	MIRO2B
RHOA_7-178	100	95.91	87.79	59.88	58.14	56.98	58.14	54.97	54.65	52.33	48.52	44.71	46.43	50	52.98	53.49	50	43.64	40.83	41.42	25.95	28.48	18.06	16.67
RHOC_7-177	95.91	100	88.89	60.23	58.48	57.89	57.89	54.39	54.39	52.63	48.52	45.29	46.43	49.4	52.38	52.63	50.88	43.9	39.29	39.88	26.11	28.66	17.53	16.13
RHOB_7-178	87.79	88.89	100	59.88	58.14	57.56	56.4	53.8	54.07	52.33	48.52	45.88	45.24	46.43	51.79	53.49	50	44.24	37.87	38.46	27.22	29.75	20	17.95
RAC1_5-176	59.88	60.23	59.88	100	95.35	95.35	73.26	73.1	66.86	63.37	60.95	57.06	41.67	45.24	47.02	52.91	51.16	44.24	43.79	43.2	25.95	27.85	20	17.95
RAC3_5-176	58.14	58.48	58.14	95.35	100	93.6	72.67	73.1	66.86	63.37	59.76	57.06	41.67	43.45	45.24	52.33	50.58	44.24	42.6	42.01	25.32	27.22	20.65	18.59
RAC2_5-176	56.98	57.89	57.56	95.35	93.6	100	73.84	71.93	64.53	62.79	59.17	55.88	41.67	44.05	44.05	49.42	52.33	43.03	42.6	42.01	25.95	27.85	18.06	17.31
RHOG_5-176	58.14	57.89	56.4	73.26	72.67	73.84	100	61.99	58.14	55.81	53.25	51.18	40.48	44.64	45.24	46.51	48.26	41.82	40.24	40.24	25.95	27.22	17.42	17.31
CDC42_5-175	54.97	54.39	53.8	73.1	73.1	71.93	61.99	100	70.76	66.67	61.54	57.06	39.29	40.48	42.26	46.2	45.03	46.34	41.07	40.48	25.48	25.48	19.48	18.06
TC10_11-182	54.65	54.39	54.07	66.86	66.86	64.53	58.14	70.76	100	84.3	56.8	57.06	41.07	42.26	44.64	44.77	48.84	43.03	40.24	40.24	27.22	24.68	19.35	18.59
TCL_23-194	52.33	52.63	52.33	63.37	63.37	62.79	55.81	66.67	84.3	100	55.03	53.53	39.88	39.88	41.67	44.77	49.42	42.42	39.05	39.64	24.68	22.78	18.71	17.31
WRCH1_51-219	48.52	48.52	48.52	60.95	59.76	59.17	53.25	61.54	56.8	55.03	100	68.64	37.5	37.5	39.29	42.01	41.42	43.21	38.55	39.16	27.74	25.81	16.45	18.3
WRCH2_33-203	44.71	45.29	45.88	57.06	57.06	55.88	51.18	57.06	57.06	53.53	68.64	100	34.52	33.93	35.71	41.18	39.41	44.17	36.9	36.9	26.28	25	16.34	18.83
RND1_15-183	46.43	46.43	45.24	41.67	41.67	41.67	40.48	39.29	41.07	39.88	37.5	34.52	100	62.13	68.64	41.07	42.26	32.92	36.97	35.15	23.38	24.03	18.54	16.45
RND2_9-177	50	49.4	46.43	45.24	43.45	44.05	44.64	40.48	42.26	39.88	37.5	33.93	62.13	100	75.15	43.45	44.64	36.65	36.36	36.36	20.78	22.08	15.89	16.45
RND3_25-193	52.98	52.38	51.79	47.02	45.24	44.05	45.24	42.26	44.64	41.67	39.29	35.71	68.64	75.15	100	42.86	47.02	39.75	36.36	35.15	25.32	25.32	20.53	18.42
RHOD_19-190	53.49	52.63	53.49	52.91	52.33	49.42	46.51	46.2	44.77	44.77	42.01	41.18	41.07	43.45	42.86	100	54.65	40.61	32.54	33.14	28.48	28.48	19.35	19.87
RHOF_21-192	50	50.88	50	51.16	50.58	52.33	48.26	45.03	48.84	49.42	41.42	39.41	42.26	44.64	47.02	54.65	100	36.97	29.59	30.18	28.48	27.22	18.71	14.74
RHOH_6-170	43.64	43.9	44.24	44.24	44.24	43.03	41.82	46.34	43.03	42.42	43.21	44.17	32.92	36.65	39.75	40.61	36.97	100	37.04	35.8	25.17	23.18	19.33	17.88
RHBT1_16-207	40.83	39.29	37.87	43.79	42.6	42.6	40.24	41.07	40.24	39.05	38.55	36.9	36.97	36.36	36.36	32.54	29.59	37.04	100	94.27	23.87	21.94	19.23	18.06
RHBT2_16-207	41.42	39.88	38.46	43.2	42.01	42.01	40.24	40.48	40.24	39.64	39.16	36.9	35.15	36.36	35.15	33.14	30.18	35.8	94.27	100	23.87	23.23	18.59	18.06
MIRO1A_6-166	25.95	26.11	27.22	25.95	25.32	25.95	25.95	25.48	27.22	24.68	27.74	26.28	23.38	20.78	25.32	28.48	28.48	25.17	23.87	23.87	100	73.29	20.42	13.99
MIRO2A_6-166	28.48	28.66	29.75	27.85	27.22	27.85	27.22	25.48	24.68	22.78	25.81	25	24.03	22.08	25.32	28.48	27.22	23.18	21.94	23.23	73.29	100	21.83	15.38
MIRO1B_416_579	18.06	17.53	20	20	20.65	18.06	17.42	19.48	19.35	18.71	16.45	16.34	18.54	15.89	20.53	19.35	18.71	19.33	19.23	18.59	20.42	21.83	100	45.68
MIRO2B_414_576	16.67	16.13	17.95	17.95	18.59	17.31	17.31	18.06	18.59	17.31	18.3	18.83	16.45	16.45	18.42	19.87	14.74	17.88	18.06	18.06	13.99	15.38	45.68	100

Supplementary Figures

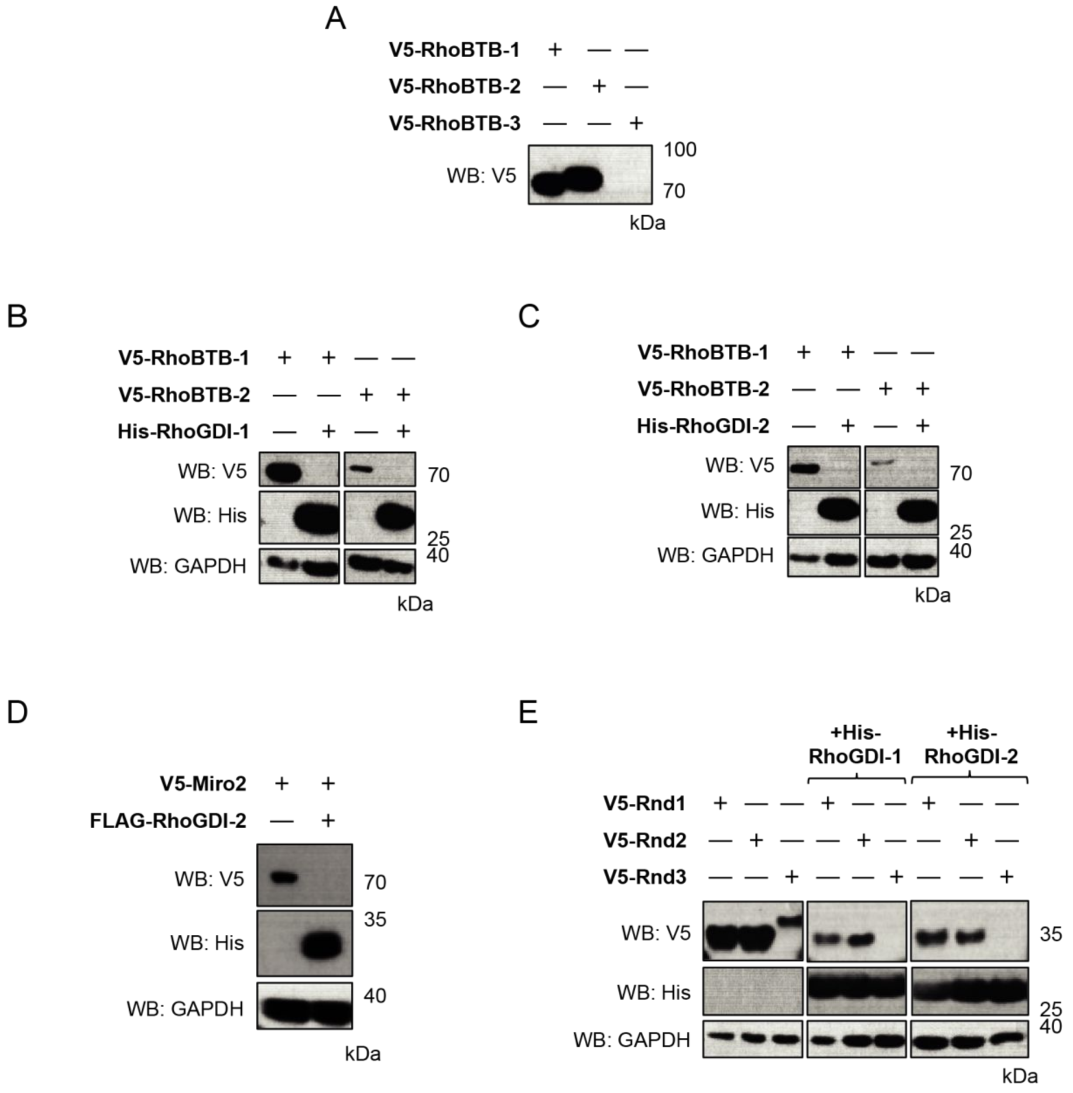

Supplementary Figure 1: The BTB subfamily, Rnd subfamily and Miro2 do not express to detectable levels in the presence of with RhoGDIs. V5-tagged expression constructs for RhoBTB1, 2 and 3, Rnd 1, 2 and 3 and Miro2 were expressed alone and with RhoGDI-1, 2 and 3 in HEK293T cells. Cells were lysed and the whole cell lysates analysed by western blotting with the appropriate antibodies. (A) RhoBTB1 and 2 express well alone, however RhoBTB-3 does not express to detectable levels. (B) RhoBTB-1 and 2 are not detected when co-expressed with RhoGDI-1. (C) RhoBTB1 and 2 were not detectable when co-

expressed with RhoGDI-2. (D) Miro2 does not express to detectable levels in the presence of RhoGDI-2. (E) Rnd1, 2 and 3 express well alone. Rnd1 and 2 expression is detectable in the presence of RhoGDI-1 and 2 but Rnd3 cannot be seen. Results are representative of at least three independent experiments.

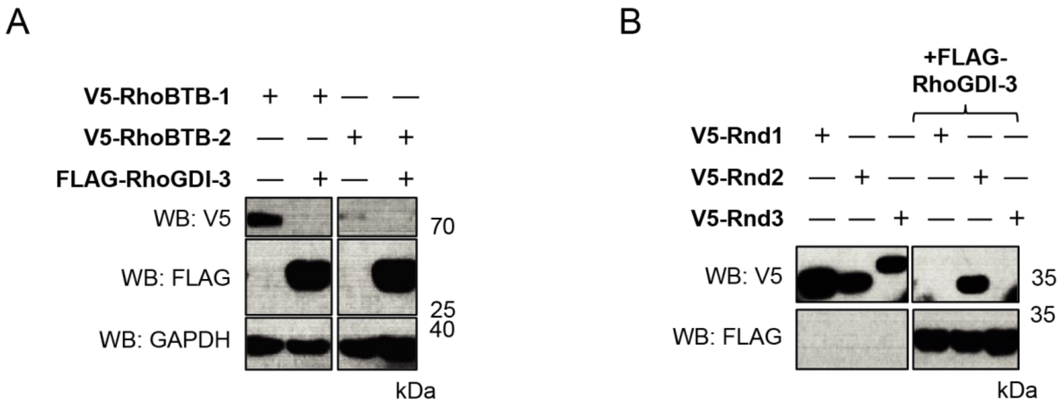

Supplementary Figure 2: Co-expression of the BTB subfamily and Rnd subfamily with RhoGDI-3. V5-tagged expression constructs for RhoBTB1 and 2, and Rnd 1, 2 and 3 were expressed alone and with RhoGDI-3 in HEK293T cells. Cells were lysed and the whole cell lysates analysed by western blotting with the appropriate antibodies. (A) RhoBTB1 and 2 express well alone but were not detected when co-expressed with RhoGDI-3. (B) Rnd1, 2 and 3 express well alone but only Rnd 2 expression is detectable in the presence of RhoGDI-3. Results are representative of at least three independent experiments.

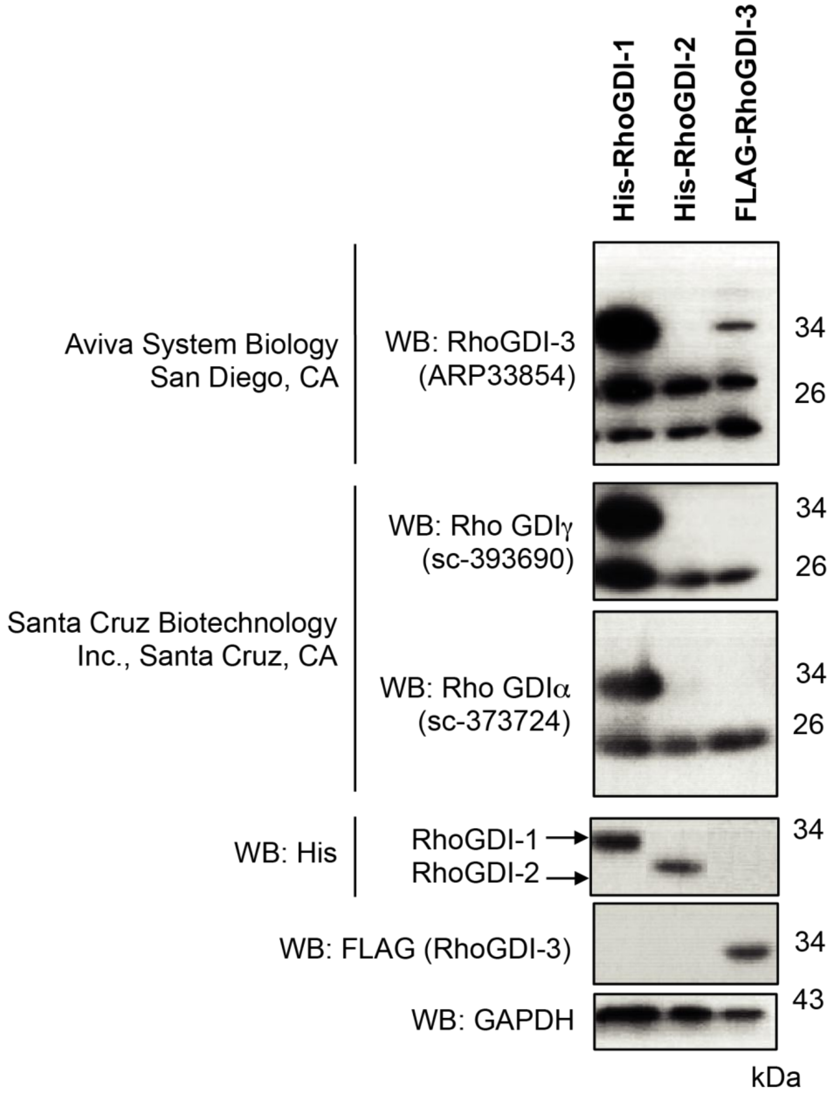

Supplementary Figure 3: Analysis of RhoGDI antibody specificity. Three commercial antibodies were tested for specificity against the three RhoGDI isoforms. His-tagged RhoGDI-1 and 2 and FLAG-tagged RhoGDI-3 were exogenously expressed in HEK293T cells and the whole cell lystaes analysed by western blotting. Expression of the proteins was identified using antibodies detecting the relevant tags (bottom panels). ARP33854 (Aviva System Biology) is a rabbit polyclonal raised to the N-terminus of RhoGDI-3 and therefore supposed to be RhoGDI-3 specific. ARP33854 detects RhoGDI-3 weakly but has much stronger cross reactivity to RhoGDI-1 (upper panel). sc-393690 (Santa Cruz Biotechnology) is a mouse

monoclonal raised to residues 1-95 of RhoGDI-3 and again supposed to be RhoGDI-3 specific. sc-393690 reacts strongly but only to RhoGDI-1. sc-373724 (Santa Cruz Biotechnology) is a mouse monoclonal raised against RhoGDI-1 and this antibody only shows reactivity to RhoGDI-1. Results are representative of at least three independent experiments.
